# A Computational Study of the Factors Influencing the PVC-Triggering Ability of a Cluster of Early Afterdepolarization-Capable Myocytes

**DOI:** 10.1371/journal.pone.0144979

**Published:** 2015-12-16

**Authors:** Soling Zimik, Alok Ranjan Nayak, Rahul Pandit

**Affiliations:** 1 Department of Physics, Centre for Condensed Matter Theory, Indian Institute of Science, Bangalore, Karnataka, India; 2 Robert Bosch Centre for Cyber Physical Systems, Indian Institute of Science, Bangalore, Karnataka, India; 3 Jawaharlal Nehru Centre for Advanced Scientific Research, Bangalore, Karnataka, India; University of Minnesota, UNITED STATES

## Abstract

Premature ventricular complexes (PVCs), which are abnormal impulse propagations in cardiac tissue, can develop because of various reasons including early afterdepolarizations (EADs). We show how a cluster of EAD-generating cells (EAD clump) can lead to PVCs in a model of cardiac tissue, and also investigate the factors that assist such clumps in triggering PVCs. In particular, we study, through computer simulations, the effects of the following factors on the PVC-triggering ability of an EAD clump: (1) the repolarization reserve (RR) of the EAD cells; (2) the size of the EAD clump; (3) the coupling strength between the EAD cells in the clump; and (4) the presence of fibroblasts in the EAD clump. We find that, although a low value of RR is necessary to generate EADs and hence PVCs, a very low value of RR leads to low-amplitude EAD oscillations that decay with time and do not lead to PVCs. We demonstrate that a certain threshold size of the EAD clump, or a reduction in the coupling strength between the EAD cells, in the clump, is required to trigger PVCs. We illustrate how randomly distributed inexcitable obstacles, which we use to model collagen deposits, affect PVC-triggering by an EAD clump. We show that the gap-junctional coupling of fibroblasts with myocytes can either assist or impede the PVC-triggering ability of an EAD clump, depending on the resting membrane potential of the fibroblasts and the coupling strength between the myocyte and fibroblasts. We also find that the triggering of PVCs by an EAD clump depends sensitively on factors like the pacing cycle length and the distribution pattern of the fibroblasts.

## Introduction

Life-threatening cardiac arrhythmias, like ventricular fibrillation (VF), are associated with the abnormal propagation of waves of electrical activation through cardiac tissue [1, 2]. The degeneration from a normal heart beat to an irregular heart beat (like in VF) can occur, *inter alia*, if there are pathological triggers, like premature ventricular complexes (PVCs) that interrupt the rhythmic propagation of electrical waves. These PVCs can develop in cardiac tissue if the myocytes are capable of triggering early afterdepolarizations (EADs), which are abnormal voltage oscillations in the repolarization phase of an action potential (AP). A cell becomes susceptible to EADs when its repolarization reserve (RR), i.e., the ability of the cell to repolarize, is reduced by increasing the inward currents, or reducing the outward currents, or both [3–5]. In tissue, EADs can cause PVCs and also lead to complex wave dynamics and patterns [6–9]. The ability of an isolated myocyte to trigger EADs depends on the conductances and kinetics of the gating variables of its ion channels. However, in tissue, if the EAD myocyte is coupled to neighboring normal myocytes, then the threshold for triggering EADs increases, because of the local source-sink mismatch. The loading effect from the neighboring normal cells drains the depolarizing currents from the EAD cell and can impede its ability to excite EADs. In order to overcome this local source-sink mismatch, a certain minimum number of EAD cells must be organized into a cluster [10]. This number depends on the RR of the cell, and the spatial dimension of the tissue; it is ≃ 80–150 cells in one dimension and progressively increases as we go to two and three dimensions [10]. Therefore, the size of the cluster of EAD cells is a factor in deciding whether the cells in the clump can maintain their EAD-triggering abilities. If an EAD clump is large enough to retain its EAD-generating ability, it can trigger abnormal excitations like PVCs, which are implicated as sources of cardiac arrhythmias [11, 12].

Apart from the size of the clump of EAD cells, other factors, like the coupling strength between the cells [10, 13] and the presence of fibroblasts [10, 14–16], can influence the PVC-triggering phenomenon. Evidence for the existence of gap-junctional coupling between fibroblasts and myocytes has been found, in cell-culture experiments [17–19]. Such coupling between the fibroblasts and myocytes can influence the EAD-triggering ability of the myocytes [10, 15, 16] and, thereby, promote PVCs. Another way in which fibroblasts can influence the EAD-triggering ability of myocytes in tissue is through fibrosis. After incidents like myocardial infarction, fibroblasts can rapidly deposit collagens during the wound-healing process, a condition known as fibrosis. Such fibrotic tissues provide a suitable network for EAD cells to trigger PVCs [14], because the interposition of collagens reduces the local source-sink mismatch. Given that a disease like heart failure is associated with fibrosis [20, 21], reduction in coupling strength [22, 23], and the promotion of EAD cells [24–27], it is important to study in detail how each of these factors promote PVCs in a cardiac tissue, or the whole heart.

Some studies have examined how the sizes of EAD clumps [10], their coupling strength [10, 13], and their interaction with fibroblasts [10, 14–16] influence the EAD-triggering ability of myocytes, and thus promote PVCs. We build on the results of these studies by carrying out extensive *in silico* investigations of the triggering of PVCs by EAD clumps in state-of-the-art mathematical models for human ventricular tissue [28, 29]. We find that an EAD clump starts triggering PVCs only after a certain threshold size, and the number N of PVCs increases with the radius R of the clump. The reduction of the coupling strength between the EAD cells also assists in triggering PVCs. We model fibrosis, i.e, the deposition of collagens by fibroblasts, by distributing inexcitable point obstacles randomly in our EAD clump; and we show that such fibrosis pattern enhances the triggering of PVCs; this enhancement increases with the percentage P_f_ of fibrosis, reaches a maximum at P_f_ ≃ 40%, and then decreases until at P_f_ ≃ 55% the EAD clump loses its ability to trigger PVCs. We also study the effects of the formation of gap-junctional coupling between myocytes and fibroblasts. We find that the gap-junctional coupling between fibroblasts and myocytes either enhances or impedes the PVC-triggering ability of the EAD clump depending on the resting membrane potential of the fibroblasts, and the coupling strength between them. The PVC-triggering ability of the EAD clumps also depends sensitively on the distribution pattern of fibroblasts.

The remaining part of this paper is organized as follows. The Section entitled **Materials and Methods** describes the models we use and the numerical methods we employ to study them. The Section entitled **Results** contains our results, from tissue-level simulations. The Section entitled **Discussions** is devoted to a discussion of our results in the context of earlier numerical and experimental studies.

## Materials and Methods

For our myocyte cell we use the O’Hara-Rudy model (ORd) for a human ventricular cell [28] with the modifications as implemented in Ref.[30], where the fast sodium current (I_Na_), of the original ORd model, has been replaced with that of the model due to Ten Tusscher and Panfilov [29] (This was actually suggested by O’Hara and Rudy themselves (see Ref. [31])). This modification is implemented because of the slow conduction velocity of the original ORd model in cardiac-tissue simulations [30]. To simulate fibrosis in our study, the collagen deposits from the fibroblasts are modelled as inexcitable point obstacles by setting the diffusion constant D = 0. The fibroblasts are modelled as passive cells, as is done in other computational studies [32, 33]; for these fibroblasts we use the model given by MacCannell, *et al.* [34]. The membrane capacitance of the fibroblasts is taken to be 6.3 pF, and their membrane conductance is 4 nS. The default gap-junctional conductance between fibroblasts and myocytes is 8 nS, unless mentioned otherwise in the text.

In a unit of a myocyte-fibroblast composite, the membrane potential of the myocyte V_m_ is governed by the ordinary differential equation (ODE)

$$\begin{array}{c} \frac{\partial V_{m}}{\partial t}=-\frac{I_{\text{ion}}+I_{\text{gap}}}{C_{m}}, \end{array}$$ (1)

where *C*_*m*_ is the myocyte capacitance, which has a value of 185 pF; *I*_ion_ is the sum of all the ionic currents of the myocyte, and *I*_gap_ is the gap-juctional current between the fibroblast and myocyte. We give *I*_ion_ and *I*_gap_ below:

$$\begin{array}{c} I_{\text{ion}}=I_{\text{Na}}+I_{\text{to}}+I_{\text{CaL}}+I_{\text{CaNa}}+I_{\text{CaK}}+I_{\text{Kr}}+I_{\text{Ks}}+I_{K1}+I_{\text{NaCa}}+I_{\text{NaK}}+I_{\text{Nab}}+I_{\text{Cab}}+I_{\text{Kb}}+I_{\text{pCa}}; \end{array}$$ (2)

$$\begin{array}{c} \;I_{\text{gap}}=G_{\text{gap}}\left(V_{m}-V_{f}\right); \end{array}$$ (3)

here V_f_ is the membrane potential of the fibroblast, and G_gap_ is the gap-junctional conductance between the fibroblast and myocyte.

A glossary of all the ionic currents of the myocyte I_ion_ is given in Table 1.

**Table 1:** Table of currents.

I_Na_	fast inward Na^+^ current
I_to_	transient outward K^+^ current
I_CaL_	L-type Ca^2+^ current
I_Kr_	rapid delayed rectifier K^+^ current
I_Ks_	slow delayed rectifier K^+^ current
I_K1_	inward rectifier K^+^ current
I_NaCa_	Na^+^/Ca^2+^ exchange current
I_NaK_	Na^+^/K^+^ ATPase current
I_Nab_	Na^+^ background current
I_Cab_	Ca^2+^ background current
I_pCa_	sarcolemmal Ca^2+^ pump current
I_Kb_	K^+^ background current
I_CaNa_	Na^+^ current through the L-type Ca^2+^ channel
I_CaK_	K^+^ current through the L-type Ca^2+^ channel

The various ionic currents incorporated in the ORd model are tabulated above. The symbols used for the currents follow Ref. [28], which gives the dependence of all these currents on the membrane potential and the equations for all the gating variables.

The membrane potential of the fibroblast is governed by the equation

$$\begin{array}{c} \frac{\partial V_{f}}{\partial t}=\frac{I_{\text{gap}}-I_{f}}{C_{f}}, \end{array}$$ (4)

where *C*_*f*_ is the membrane capacitance of the fibroblast, and *I*_f_ is the fibroblast current,

$$\begin{array}{c} \;I_{f}=G_{f}\left(V_{f}-E_{f}\right); \end{array}$$ (5)

here G_f_ is the membrane conductance of the fibroblast, and E_f_ is the resting membrane potential of the fibroblast.

The spatiotemporal evolution of the membrane potential (V_m_) of the myocytes in tissue is governed by a reaction-diffusion equation, which is the following partial-differential equation (PDE):

$$\begin{array}{c} \frac{\partial V_{m}}{\partial t}+\frac{I_{\text{ion}}+I_{\text{gap}}}{C_{m}}=D\nabla^{2}V_{m}, \end{array}$$ (6)

where D is the diffusion constant between the myocytes. I_gap_ = 0 if no fibroblast is attached to the myocyte.

### Numerical Methods

**Fig 1:**
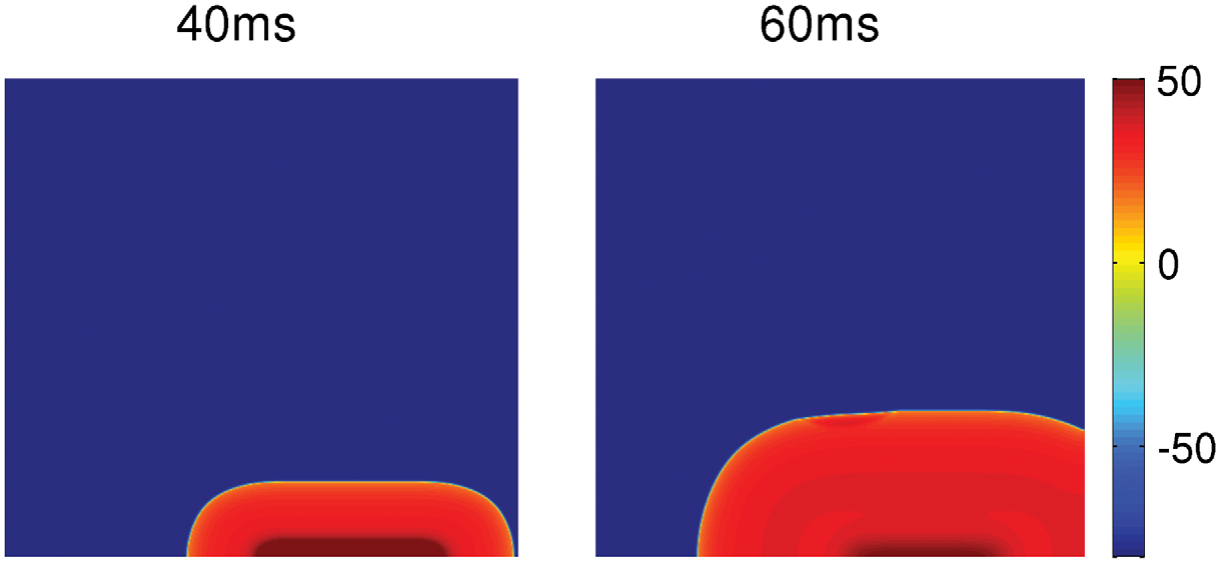
Asymmetric pacing protocol. Pseudocolor plots showing the spatiotemporal evolution of V_m_ and depicting the pacing protocol we use for our 2D simulation. The stimulus is applied over a region of 0.14x3 cm^2^ on the lower-right boundary of the domain. The colorbar indicates the value of V_m_ in mV.

We use a forward-Euler method to solve the ODEs Eqs (1) and (4) for V_m_ and V_f_, respectively, and also for the ODEs for the gating variables of the ionic currents of the myocyte. For solving the PDE Eq (5), we use the forward-Euler method for time marching with a five-point stencil for the Laplacian. We set D = 0.0012 cm^2^/ms. The temporal and spatial resolutions are set to be *δx* = 0.02 cm and *δt* = 0.02 ms, respectively. The conduction velocity in the tissue, with the above set of parameters, is 65 cm/s. In our two-dimensional (2D) tissue simulations, we use a domain size of 448 × 448 grid points, which translates into a physical size of 8.96 × 8.96 cm^2^. All our 2D simulations are carried out for a duration of 15 seconds. For pacing the tissue, we use an asymmetric pacing protocol, in which an external stimulus is applied over a small region (0.14 ×3 cm^2^) on the lower boundary of the domain as shown in Fig 1. The strength and duration of the stimulus of this asymmetric pulse are -150 *μ*A/*μ*F and 3 ms, respectively.

## Results

We now present the results of our studies of the factors that are important in triggering PVCs by an EAD clump. We first elucidate the effects of the repolarization reserve (RR) on PVCs. We then investigate the effects of the size of EAD clumps on PVCs. We follow this with a study of the effects of the coupling strengths on PVC triggering. We then investigate how the presence of fibroblasts modulate the triggering of PVCs. Finally, we present a few representative results to show how a spiral wave interacts with an EAD clump.

### Role of Repolarization reserve in triggering PVCs

**Fig 2:**
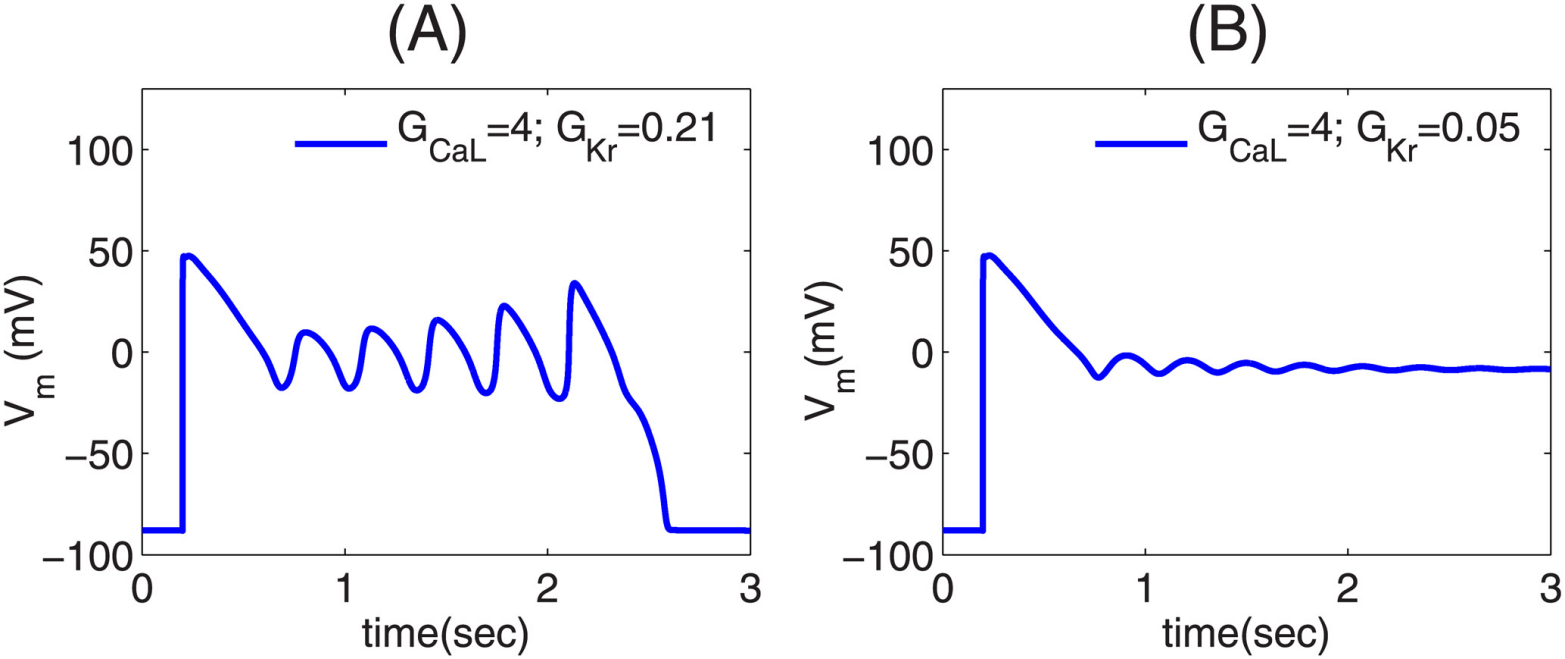
Plots of V_m_ versus time illustrating the two types of APs. (A) Type-I AP with the EAD oscillations increasing with time until the cell repolarizes to the resting membrane potential; (B) Type-II AP, with decaying EAD oscillations, which relax to a higher potential than the normal resting membrane potential. The parameter sets of the APs are given in the top-right corners of the figures.

The repolarization reserve (RR) is defined as the ability of the cell to repolarize after it is depolarized. Therefore, if we reduce RR either by enhancing inward currents, like I_CaL_, or by decreasing outward currents, like I_Kr_, we can induce EADs. Although the presence of EADs in the myocytes promotes the triggering of PVCs, not all action potentials (APs) with EADs lead to PVCs. Fig 2(A) and 2(B) show two types of APs with EADs. These EADs are induced by increasing the conductance of I_CaL_ (G_CaL_) and decreasing the conductance of I_Kr_ (G_Kr_). The parameter sets for the two AP types are given in the top-right corners of the figures. Here the values of G_CaL_ and G_Kr_ indicate the multiples of their control values. Hence, G_CaL_ = 4 and G_Kr_ = 0.21 denote, respectively, that we use a value of G_CaL_(G_Kr_) that is 4 (0.21) times its control value. We refer to the APs of Fig 2(A) and 2(B) as type-I and type-II APs, respectively. The value of G_CaL_ is the same in both the AP types, but the value of G_Kr_ is lower for the type-II AP, and hence the RR in the type-II AP is lower than that of the type-I AP. In the type-I AP (see Fig 2) the EAD oscillations have an amplitude that increases with time until the AP repolarizes to its resting value. By contrast, the type-II AP has decaying EAD oscillations, which relax to a potential higher than the normal resting membrane potential. (Type-II APs, with repolarization failure, are not just a result of computational models, but, they are also seen in experiments [35].) The amplitude of the EAD oscillations in the type-II AP is lower compared to that for the type-I AP.

**Fig 3:**
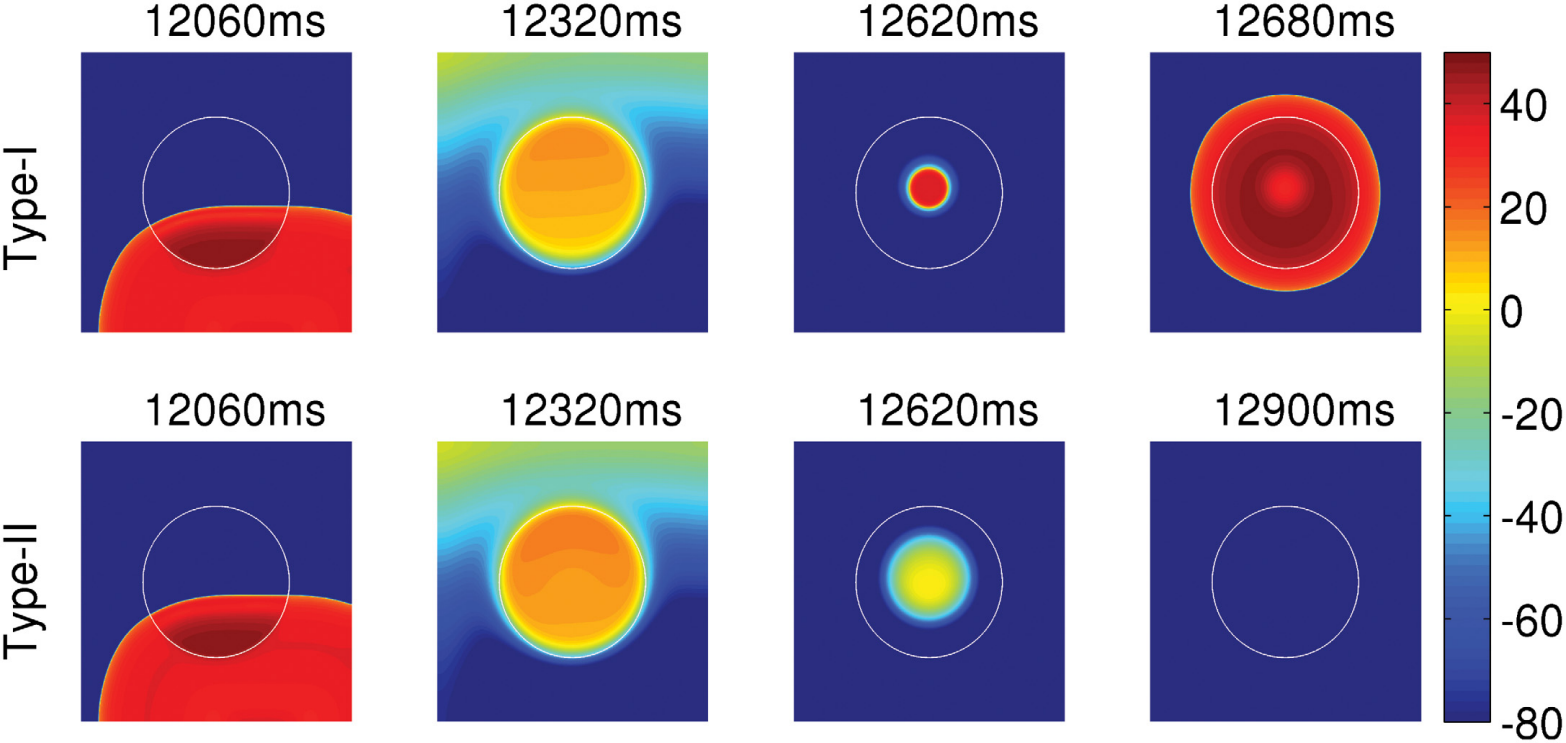
The dependence of PVCs on the repolarization reserve (RR). Pseudocolor plots of V_m_ showing its spatiotemporal evolution, when the tissue is paced at PCL = 1000 ms with EAD clumps of radius R = 2.4 cm, but two different types of APs: Type-I AP (top panel) and type-II AP (bottom panel). The white circular contour marks the periphery of the EAD clump, where the cells inside and outside the contour are EAD and normal cells, respectively. The clump with a type-I AP triggers PVCs, whereas the clump with a type-II AP does not trigger PVCs.

We now consider two clumps; one of these yields APs of type-I and the other APs of type-II. The clumps are circular and are of the same radius R = 2.4 cm. The circular clumps are embedded in the middle of our simulation domain and we then pace the domain by using asymmetric pulses at a pacing cycle length (PCL) of 1000 ms. Fig 3 shows the sequence of pseudocolor plots of V_m_ that we obtain at different times as we pace the tissue. The white circular contour in Fig 3 marks the periphery of the EAD clump. The clump with a type-I AP (top panel) triggers PVCs, whereas the clump with a type-II AP (bottom panel) does not trigger PVCs (see S1 Video). Even if we increase R, a clump with type-II APs does not lead to PVCs: although type-II APs arise from a lower RR than do type-I APs, the amplitude of EAD oscillations for the former are too weak to excite cells that lie near the EAD-clump boundary.

### Effect of the size of EAD clumps on PVC triggering

**Fig 4:**
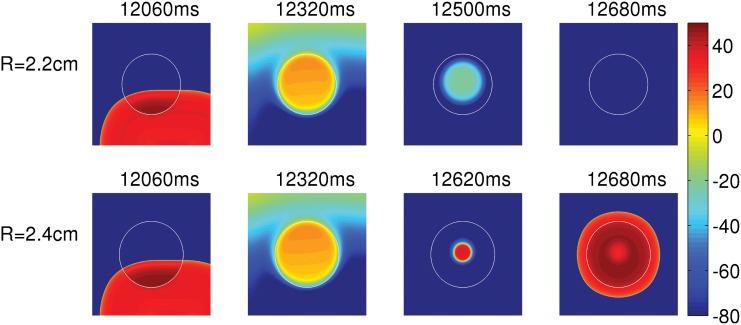
The dependence of the triggering of PVCs on the size R of an EAD clump. Pseudocolor plots of V_m_, which shows that the clump with radius, R = 2.2 cm (top panel) does not trigger PVCs, whereas the bigger clump with R = 2.4 cm (bottom panel) triggers PVCs.

**Fig 5:**
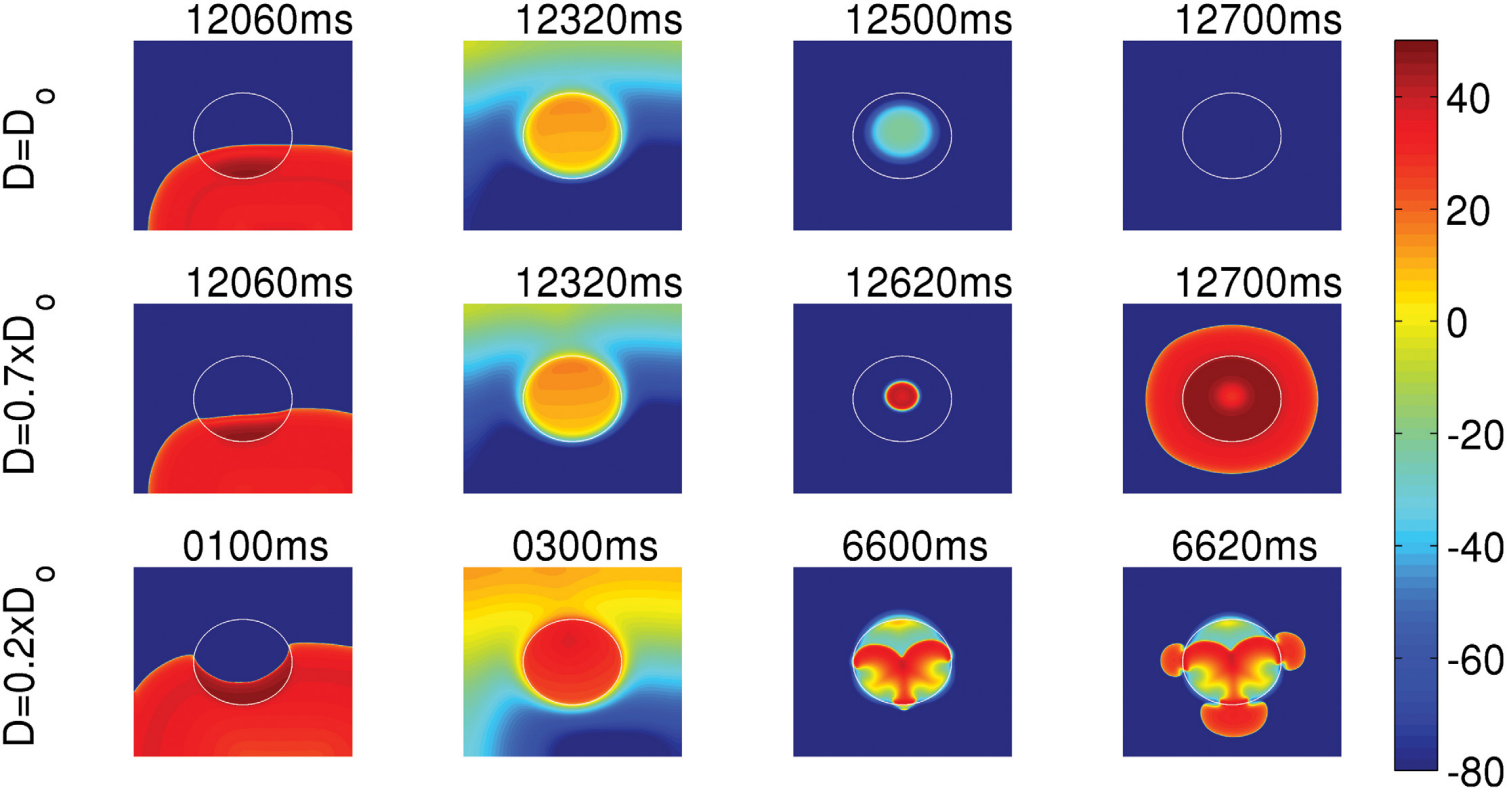
Dependence of the number N of PVC triggerings on the radius R, normalized coupling strength D/D_o_, and the percentage of fibrosis P_f_. The blue and red curves are for PCL = 1000 ms and 1400 ms, respectively. (A) Plots of N versus R, which show that the number of PVCs increases roughly with R. (B) Plots of N versus D/D_o_. The number of PVC triggerings increases initially with the reduction of D/D_o_, but saturates roughly when D/D_o_ ≲ 0.4 and ≲0.65 for PCL = 1000 and 1400 ms, respectively. (C) Plots of N versus the percentage of fibrosis P_f_ show a maximum at P_f_ ≃ 40%. A comparison of the plots in (A), (B), and (C) for the two different values of PCL, namely, PCL = 1000 ms and PCL = 1400 ms, shows that these plots depend sensitively on PCL.

We explore the dependence of PVC triggering on the radius R of an EAD clump for type-I APs. Henceforth we refer to the type-I EAD cells as EAD cells. We pace the tissue by using asymmetric pulses at a PCL of 1000 ms. Fig 4 shows the sequence of pseudocolor plots of V_m_ at different times when we pace the tissue in the presence of EAD clumps, for two different radii R = 2.2 (top panel) and R = 2.4 cm (bottom panel). This figure shows that the small clump (R = 2.2 cm) does not trigger PVCs, but the large one (R = 2.4 cm) does (see S2 Video). The number of PVCs increases roughly with the size R of the clump as shown in Fig 5(A). In Fig 5(A), we plot the number N of PVCs, triggerd within the 15 seconds of our simulation time, versus R for PCL = 1000 ms (blue curve) and PCL = 1400 ms (red curve). These plots show that N increases, roughly, with R; but the details of the plots in Fig 5 depend on PCL, which is because of the rate dependence of EADs (see, e.g., Ref.[9]); and the non-monotonic and “noisy” behavior of N as a function of R or PCL is a manifestation of the sensitive dependence on parameter values because of the underlying spatiotemporal chaos in our extended dynamical system (this sensitive dependence on parameter values has also been shown extensively, in studies of inhomogeneities in mathematical models for cardiac tissue, in Refs [36, 37]). Even the threshold size of the EAD clump, after which it triggers PVCs, depends on factors including the PCL and parameter set we use for the EAD cells.

### Effect of coupling strength in triggering PVCs

**Fig 6:**
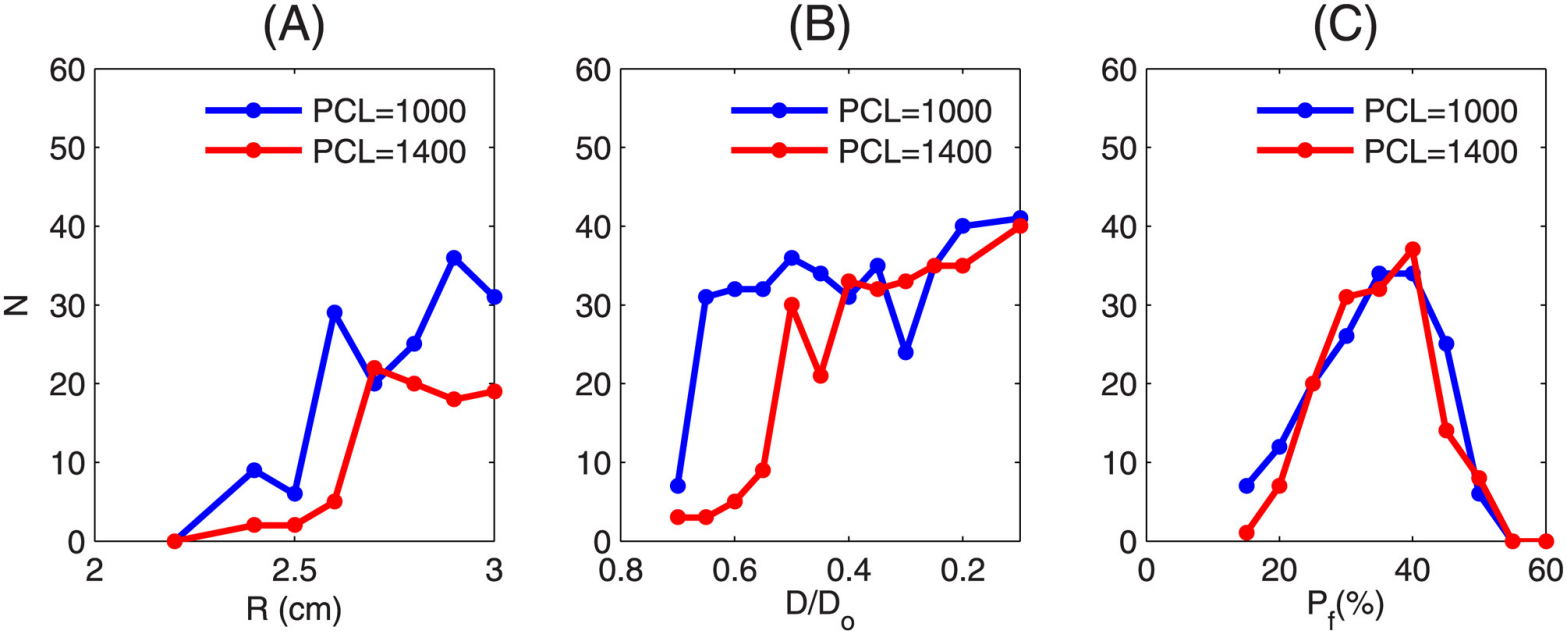
The dependence of the triggering of PVCs on the coupling strength of the EAD clump. The clumps in the top, middle, and the bottom panels have radii R = 2 cm. The clump with D = D_o_ (top panel) does not trigger PVCs, whereas the clumps with D = 0.7xD_o_, (middle panel) and D = 0.2xD_o_ (bottom panel) trigger PVCs. The clump with D = 0.2xD_o_ supports small-wavelength spirals inside the clump as shown at times 6600 ms and 6620 ms.

Here we show how the reduction in the coupling strength, which we achieve by reducing the diffusion constant between the EAD cells, assists in triggering PVCs. We take EAD clumps of the same radius R = 2 cm, but with different values of the diffusion constants D inside the clump. In Fig 6 (top panel) D inside the clump is equal to its normal value D_o_ = 0.0012 cm^2^/s; and in the middle and bottom panels the Ds are reduced to 0.7 and 0.2 times the normal value. The diffusion constant outside the clump is always set to the normal value D_o_. The clump with D = D_o_ (top panel) does not trigger PVCs, but the ones with reduced Ds (middle and bottom panels) triggers PVCs (see S3 Video). Thus, reducing the coupling strength between the EAD cells facilitates the triggering of PVCs. The reason is as follows. If the coupling strength is high, the depolarizing currents of the EAD cells at the periphery of the clump are quickly drained to the neighboring normal cells; and only a few cells lying in the bulk of the clump retain their ability to excite EADs, which is not enough to overcome the source-sink mismatch to trigger PVCs. However, if the coupling strength is reduced, because the cells are weakly coupled, more cells in the clump retain their ability to produce EADs and, therefore, can trigger PVCs. If the coupling strength in the clump is extremely reduced, as in the bottom panel (D = 0.2xD_o_), the clump supports waves of small wavelengths and hence small-wavelength spirals develop inside the clump; this increases the PVC-triggering rate. The existence of such mini spirals, inside a region of ionic inhomogeneity, with a low value of D, has also been seen in experiments [38]. Fig 5(B) shows the number N of PVCs for different values of D for PCL = 1000 ms and PCL = 1400 ms, which are indicated by blue and red curves, respectively. The diffusion constant on the horizontal axis is normalized by its normal value (D_o_ = 0.0012 cm^2^/s). This plot shows that N increases as D is reduced and almost saturates for low values of D. Note that N depends sensitively on PCL.

### Effects of fibroblasts on PVC triggering

We now present the effects of fibroblasts on the triggering of PVCs by an EAD clump.

**Fig 7:**
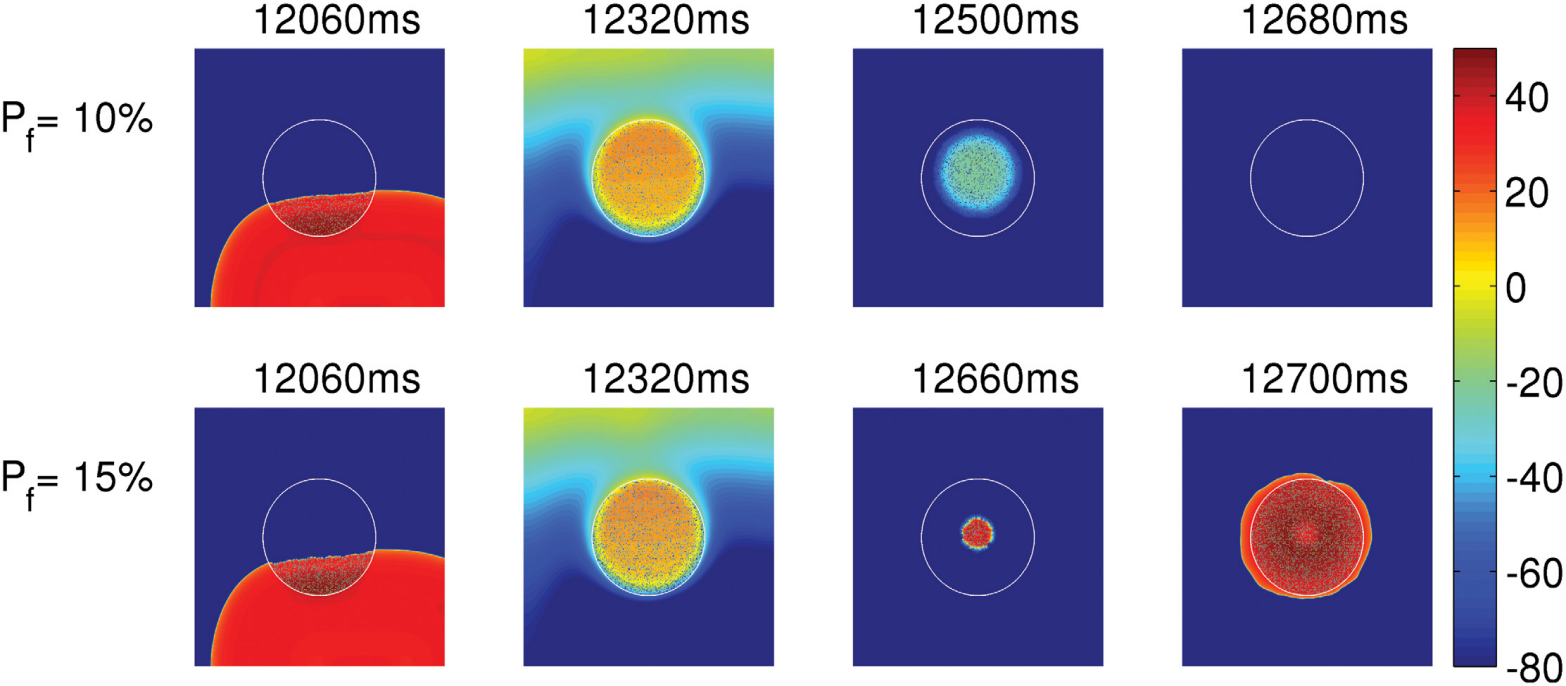
The dependence of PVC triggerings on the degree of fibrosis. Pseudocolor plots of V_m_ illustrating the triggering of PVCs with 15% fibrosis (bottom panel) and the absence of PVCs with 10% fibrosis (top panel) in an EAD clump with radius R = 2cm.

**Fibrosis and PVCs:** We show how the interruption of the coupling between EAD myocytes by collagen deposits, simulated here as inexcitable point obstacles, facilitates the triggering of PVCs. We populate the EAD clump of radius R = 2 cm with point obstacles in a diffuse pattern [33, 39, 40]. Fig 7 depicts the pseudocolor plots of V_m_ at different times for the cases when EAD clumps with P_f_ = 10% (top panel) and P_f_ = 15% (bottom panel) are paced with PCL = 1000 ms. Here P_f_ indicates the percentage of sites at which we place the inexcitable point obstacles. Henceforth we refer to P_f_ as the percentage of fibrosis. The clump with 15 percent fibrosis triggers PVCs, whereas the one with 10 percent fibrosis does not (see S4 Video). The number of PVCs increases with P_f_ up to 40%, as shown in Fig 5(C), when we use PCL = 1000 ms (blue curve) and PCL = 1400 ms (red curve). This increase in the number of PVCs is because, as P_f_ increases, the mean number of neighboring myocytes reduces, which in turn lowers the local source-sink mismatch, and thus promotes PVCs. However, after P_f_ = 40%, PVC triggering decreases, because there is a trade-off between P_f_, which reduces the source-sink mismatch, and the number of EAD cells required for the triggering of PVCs. The number of PVCs drops to zero at P_f_ = 55% for both PCL = 1000 ms and PCL = 1400 ms.

**Fig 8:**
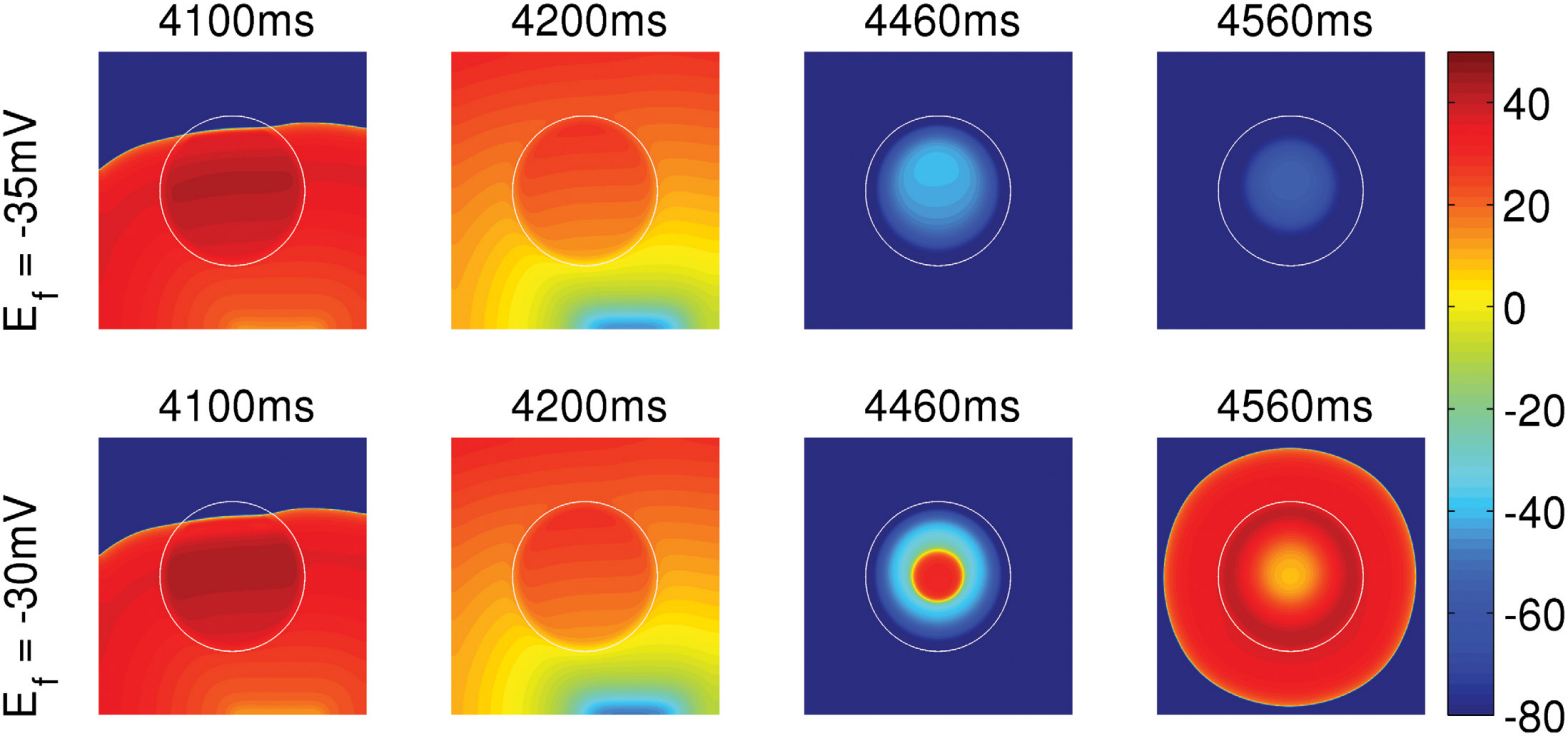
The dependence of PVC triggering on E_f_ of the fibroblasts in an EAD myocyte-fibroblast clump. Pseudocolor plots of V_m_ showing that an EAD myocyte-fibroblast clump, with R = 2.2 cm, triggers PVCs if E_f_ = -30 mV (bottom panel), but is unable to trigger PVCs if E_f_ is reduced to -35 mV (top panel).

**Fig 9:**
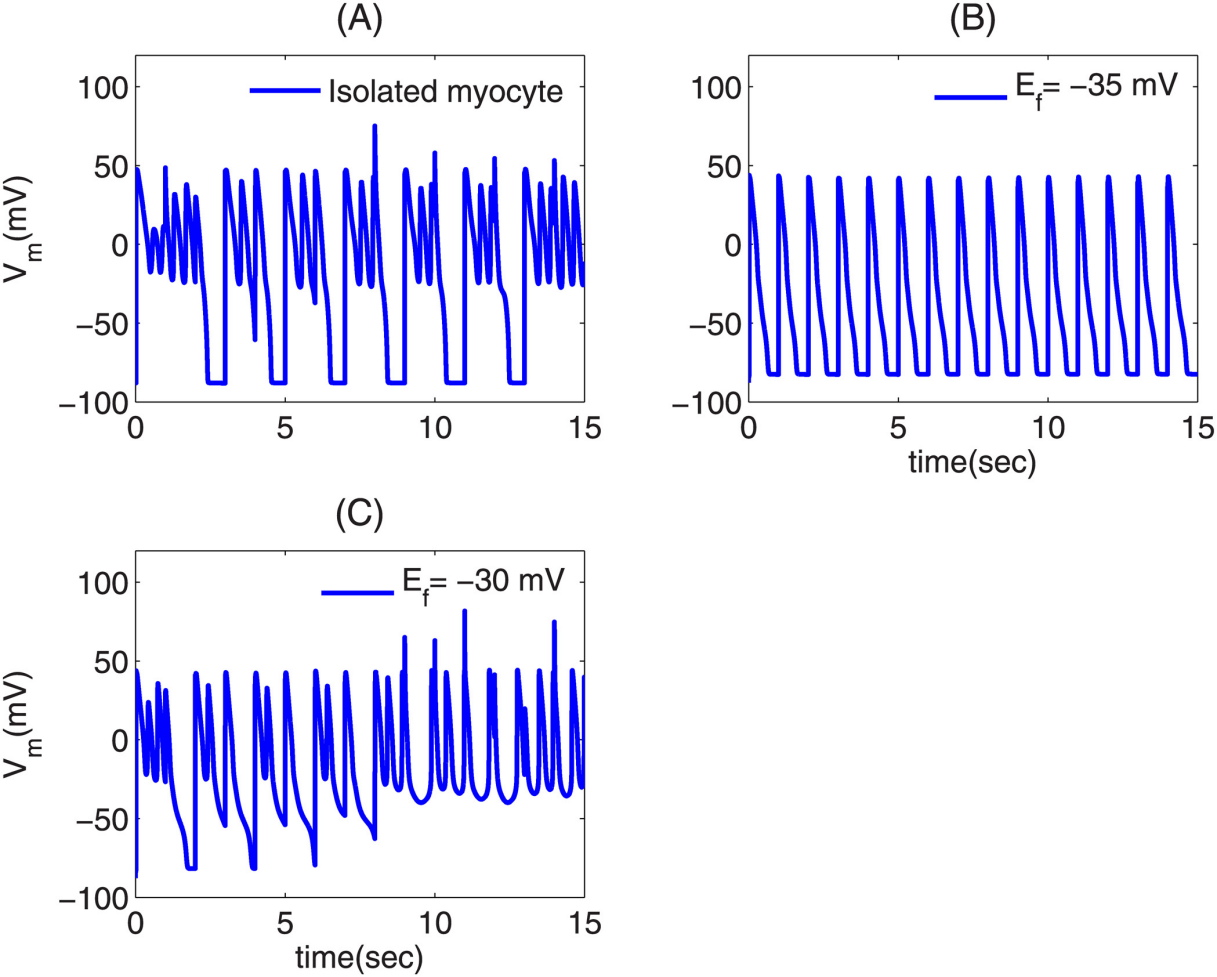
Time series of V_m_ showing the suppression and enhancement in the EAD oscillations of an EAD myocyte attached to a fibroblast. (A) an isolated EAD cell; (B) an EAD myocyte attached to a fibroblast with E_f_ = -35 mV; (C) an EAD myocyte attached to fibroblast with E_f_ = -30 mV. The myocyte is paced with a PCL = 1000ms.

**Effects of myocyte-fibroblast coupling on PVC triggering:** Fibroblasts can form heterocellular couplings with myocytes and influence the EAD-triggering ability of EAD cells, and can, thereby, modulate the triggering of PVCs. To investigate this, we take an EAD clump of radius R = 2.2 cm, which does not trigger PVCs (cf. Fig 4), and attach each of the EAD cells in the clump with a fibroblast. This clump is, therefore, a layer of EAD myocytes with a layer of fibroblasts on top of it as in Ref. [32]. As before, we pace the tissue at PCL = 1000ms. If the EAD clump is attached with fibroblasts that have a resting membrane potential E_f_ = -30 mV, the clump triggers PVCs as shown in Fig 8 (bottom panel). However, if E_f_ is reduced to -35 mV, the clump does not trigger PVCs (top panel) [see S5 Video]. This can be understood by examining the effect of E_f_ on an EAD myocyte-fibroblast composite. Fig 9(A), 9(B) and 9(C) show the V_m_ of an isolated EAD myocyte, an EAD myocyte coupled to a fibroblast with E_f_ = -35 mV, and -30 mV, respectively, with PCL = 1000 ms. The APs of the EAD myocyte, attached to a fibroblast with E_f_ = -30 mV (Fig 9(C)), show an enhancement in the EAD oscillations as compared to the APs of an isolated EAD myocyte in the sense that the last AP in Fig 9(C) shows non-decaying EAD oscillations, unlike the ones in Fig 9(A), which always repolarize. This implies that the coupling of an EAD myocyte to a fibroblast, of E_f_ = -30 mV, enhances the EAD oscillations, and hence increases the PVC-triggering ability of the EAD clump. By contrast, in Fig 9(B), the fibroblast with E_f_ = -35 mV suppresses the EADs of the myocyte and, therefore, subdues the PVC-triggering ability of the EAD clump. Thus, the PVC-triggering ability of an EAD clump, attached to fibroblasts, strongly depends on the E_f_ of the fibroblasts: the ability of the clump to trigger PVCs may either be enhanced (at high values of E_f_) or suppressed (at low values of E_f_).

**Fig 10:**
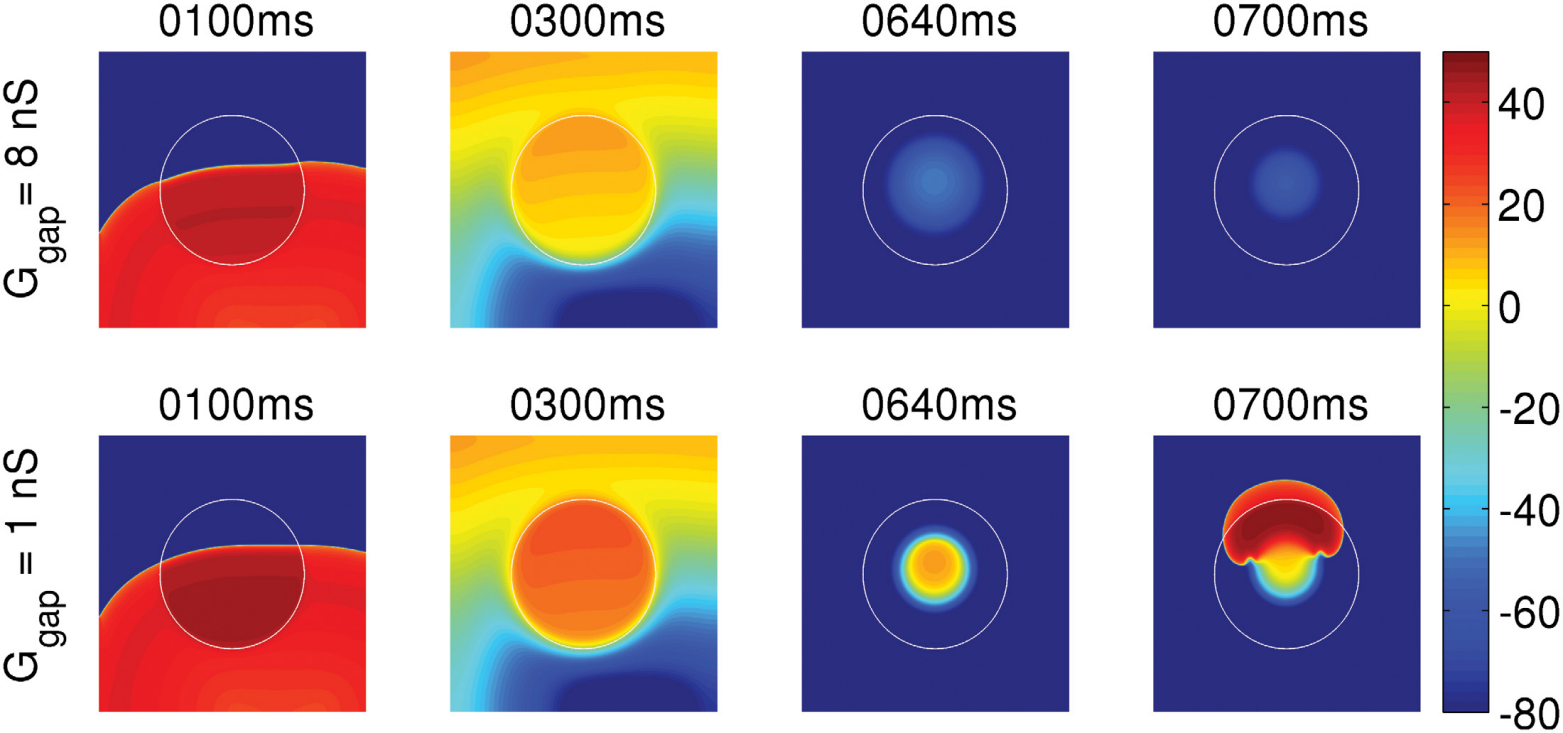
The dependence of PVC triggering on G_gap_. Pseudocolor plots of V_m_ showing that the EAD clump, with radius R = 2.4 cm, loses its ability to trigger PVCs when coupled to fibroblasts of E_f_ = -35 mV and G_gap_ = 8 nS (top panel), but retains its ability to trigger PVC, if G_gap_ is reduced to 1 nS (bottom panel).

The other factor that regulates the PVC-triggering ability of an EAD clump is the gap-junctional coupling strength G_gap_ between the myocyte and the fibroblast. In an EAD myocyte-fibroblast composite, G_gap_ modulates the amount of influence of the fibroblasts on the EAD myocyte. So, if G_gap_ is high, the fibroblast may either assist or impede the EAD-triggering ability of the EAD myocyte depending on the value of E_f_; however, if G_gap_ is low, the fibroblast does not influence the electrophysiology of the EAD myocyte significantly, and the EAD myocyte retains its ability to trigger EADs. Therefore, a PVC-triggering clump, which does not trigger PVCs when coupled strongly (high G_gap_) to fibroblasts of low E_f_, may regain its ability to trigger PVC if G_gap_ is reduced. To demonstrate this, we take an EAD clump of R = 2.4 cm, which triggers PVCs without the presence of fibroblasts (cf. Fig 4, bottom panel). We first attach the clump with fibroblasts of E_f_ = -35 mV with G_gap_ = 8 nS, and observe that the PVC triggering ability of the clump is suppressed, as shown in Fig 10, top panel. But if we reduce the G_gap_ to 1 nS, the clump regains its ability to trigger PVCs as shown in Fig 10, bottom panel (see S6 Video). Thus, given a value of E_f_, G_gap_ controls the level of influence of the fibroblast on the myocyte in an EAD myocyte-fibroblast composite and hence modulates the PVC-triggering ability of the EAD clump.

**Fig 11:**
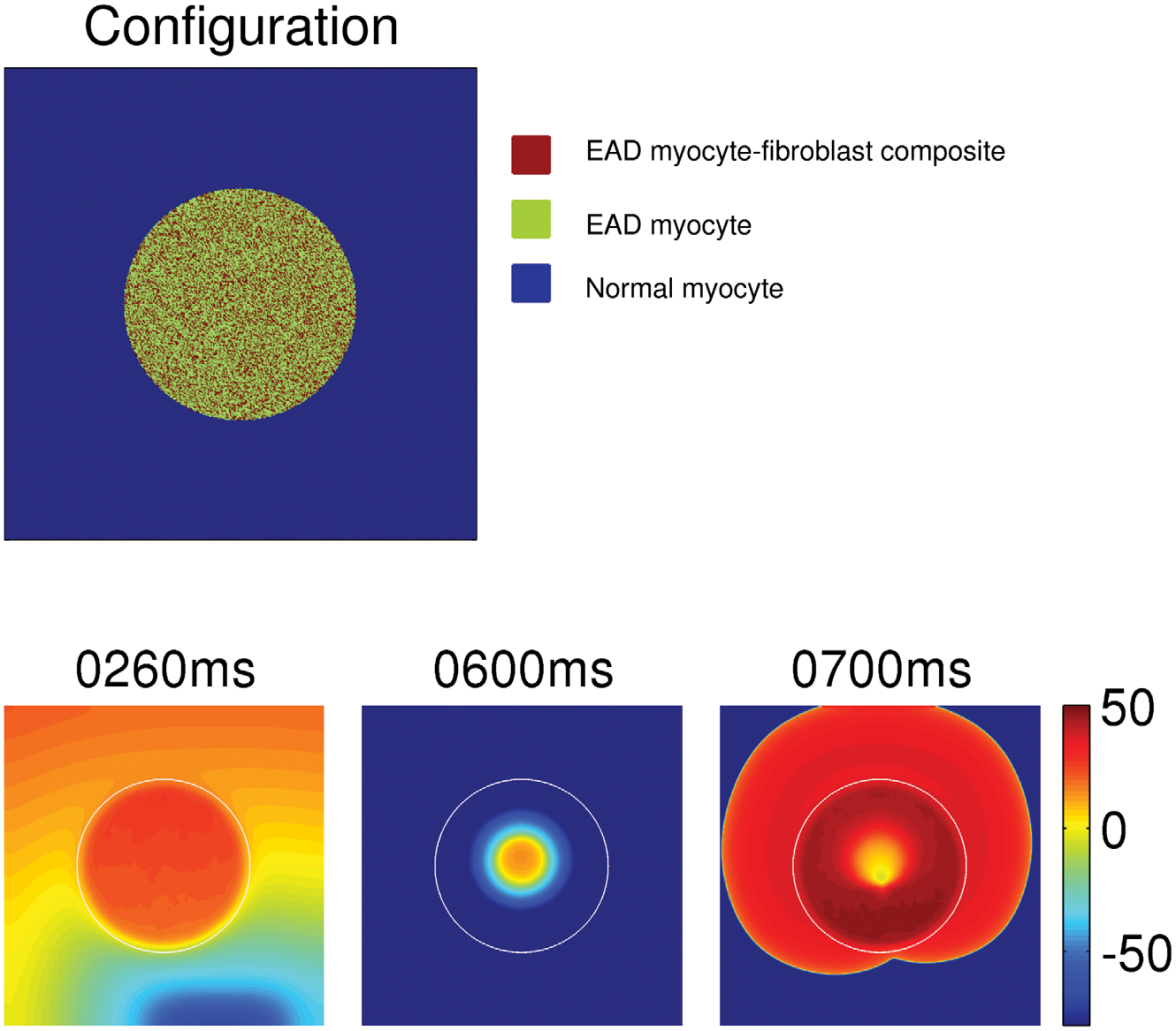
The triggering of PVCs by an EAD clump with randomly attached fibroblasts. The top panel shows the distribution of 40% of fibroblasts on the clump, where the red, green, and blue indicate, respectively, an EAD myocyte-fibroblast composite, an EAD myocyte, and a normal myocyte. The bottom panel shows the triggering of PVCs by the clump with the above configuration of fibroblasts.

**Effects of randomly attaching fibroblasts to an EAD clump:** We have, so far, employed an EAD myocyte-fibroblast bilayer clump in which a uniform layer of fibroblasts is placed atop a layer of EAD myocytes. We now explore a similar bilayer clump but with a random array of fibroblasts in the top layer. We study such a model because, in a diseased heart, the distribution of fibroblasts is random [41]; furthermore, the density of fibroblasts depends on the age of a patient [42]. To investigate the effects of inhomogeneously distributed fibroblasts, we carry out an illustrative simulation of an EAD clump with radius R = 2.2 cm, on top of which we have a layer of fibroblasts distributed randomly. The percentage P_a_ of the fibroblasts is 40%, i.e., in the top layer only 40% of the sites are occupied by fibroblasts. We show this random array of fibroblasts in the top panel of Fig 11, in which brown, green, and blue colors indicate, respectively, EAD myocyte-fibroblast composites, EAD myocytes, and normal myocytes. This clump, attached randomly to fibroblasts with E_f_ = -35 mV, triggers PVCs as shown in Fig 11 (bottom panel) when we pace with PCL = 1000 ms. This triggering of PVCs by the clump is counter-intuitive, because this value of E_f_ = -35 mV suppresses EADs as shown in Fig 9(C), and hence should prohibit the triggering of PVCs as in Fig 8 (top panel). This result implies that the inhomogeneous distribution of fibroblasts plays a role in triggering PVCs.

**Fig 12:**
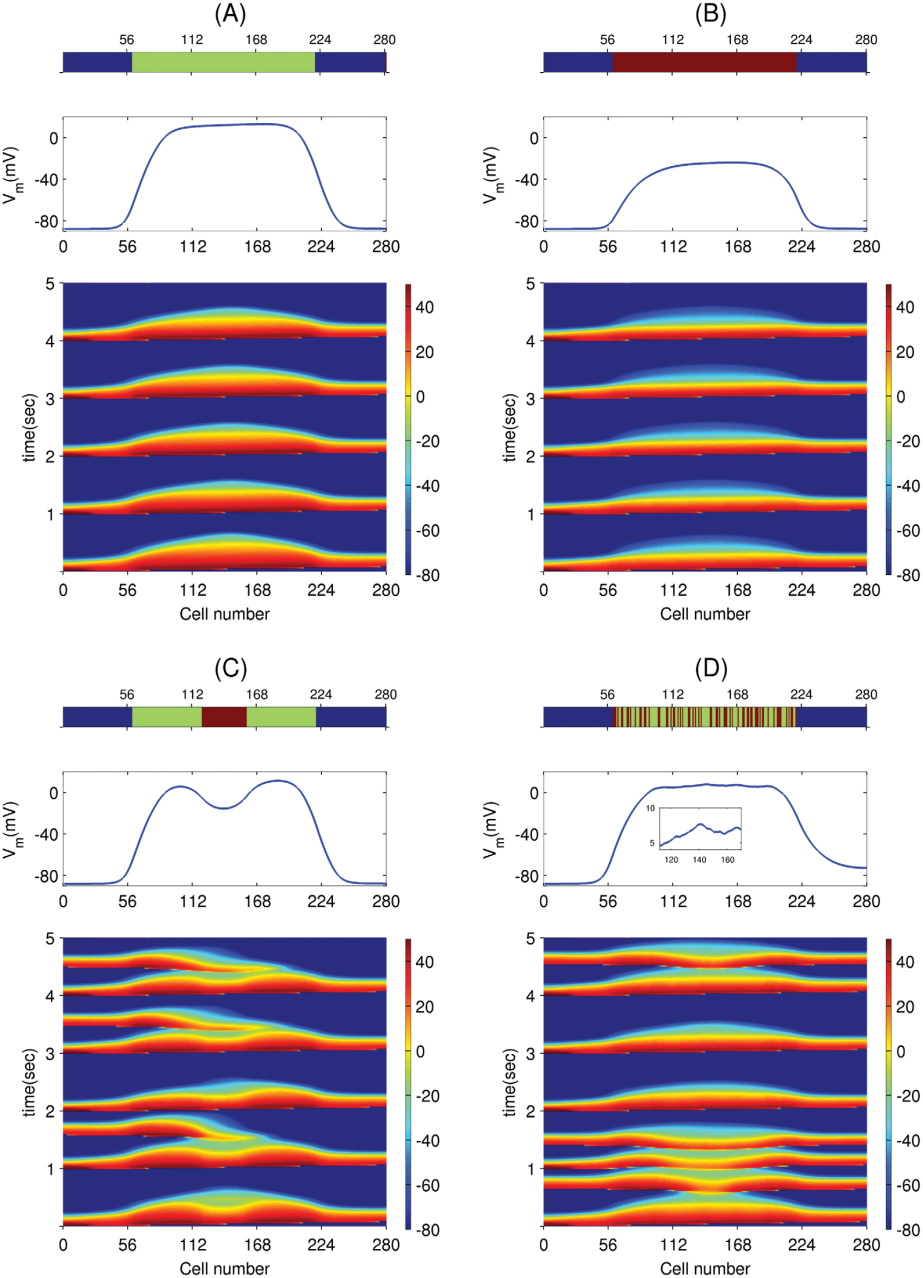
PVC triggering depends on the EAD myocyte-fibroblast pattern. The top panels of (A), (B), (C), and (D) show the distribution of fibroblasts in a one dimensional cable of 280 cells, where blue, green, and brown indicate normal myocyte, EAD myocyte, and EAD myocyte-fibroblast composites, respectively. The panels in the second row show V_m_ along the cables 20 ms after the passage of a wave. The panels in the third row show the space-time plots of V_m_ in the cables, when the cable is paced with PCL = 1000 ms from the left-side end of the cables. The patterns (C) and (D) trigger PVCs, whereas the patterns (A) and (B) do not.

To study the dependence of PVC triggering on the spatial distribution of fibroblasts, we take a one-dimensional cable of 280 myocytes and study the four different EAD myocyte-fibroblast distribution patterns, which are shown in the top panels of Fig 12(A), 12(B), 12(C) and 12(D). The blue, green, and brown colors indicate, respectively, normal myocytes, EAD myocytes, and EAD myocyte-fibroblast composites. Pattern (A) has an EAD segment of 160 EAD myocytes (60 ≤ x ≤ 220) sandwiched between normal myocytes; pattern (B) has fibroblasts homogeneously attached to the EAD segment; pattern (C) has 40 fibroblasts attached to the middle myocytes (120 ≤ x ≤ 160) in the EAD segment; and pattern (D) has fibroblasts attached randomly to the myocytes in the EAD segment. The fibroblasts have an E_f_ = -35 mV.

**Fig 13:**
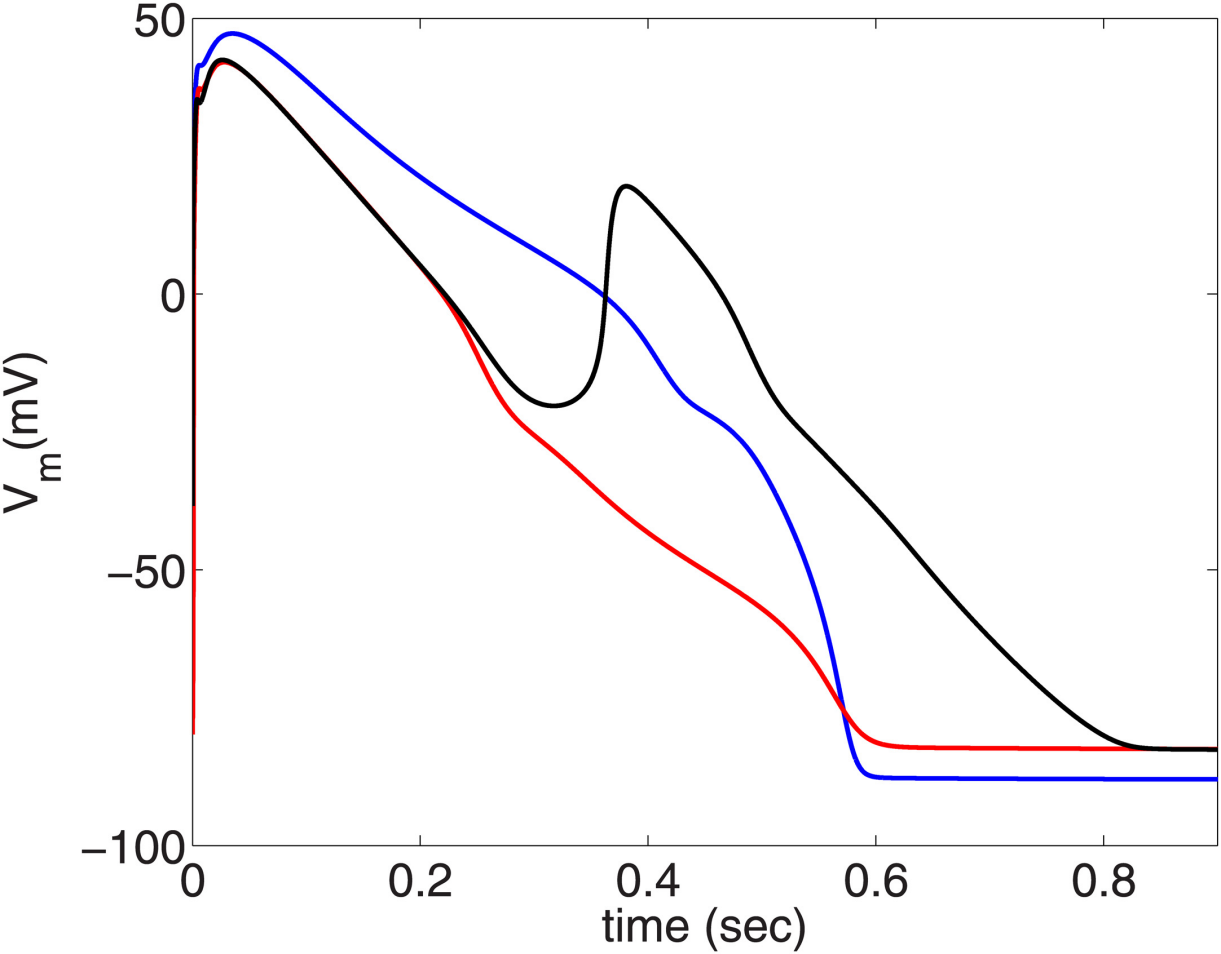
Action-potential plots showing the enhancement in the EAD depending on the EAD myocyte-fibroblast pattern (Fig 12). Plots of V_m_ versus time showing the three action potentials recorded from the middle myocyte of the EAD segment (cell number 140) for three different configurations of the cable: the blue AP is for the case where the EAD segment has no fibroblasts attached to it (Fig 12(A)); the red AP arises when the EAD segment is homogeneously coupled to fibroblasts (Fig 12(B)); and the black AP is obtained when the fibroblasts are attached to the middle of the EAD segment (Fig 12(C)).

When we pace the cable on the left-side end with PCL = 1000 ms, patterns (A) and (B) do not trigger PVCs, whereas patterns (C) and (D) trigger PVCs as shown in the space-time plots in the panels in the third rows of Fig 12(A)–12(D). The panels in the second rows of Fig 12(A)–12(D) show plots along the cable of V_m_ that remains 20 ms after the passage of a wave. As can be seen in Fig 12(C), V_m_ has a local minimum in the region where the fibroblasts are attached; this is because of the fibroblasts acting as sinks of currents from the myocytes. Such a local minimum of V_m_ in the EAD segment are absent in patterns (A) and (B) because of the homogeneous distribution of EAD myocytes and EAD myocyte-fibroblasts composites, respectively. The plot of V_m_ versus the cell number in the middle panel of Fig 12(D) seems to have a single broad plateau. However, if we magnify this plot (inset of this panel), we see that this plateau is rugged in the sense that it shows many sharp peaks and valleys, which are associated with the nonuniform distribution of fibroblasts (top panel of Fig 12(D)). The presence of such local minima in the V_m_ of the EAD segment favors the formation of EADs and, thereby, PVCs. To illustrate this, we show, in Fig 13, three plots of action potentials (APs) recorded from the central myocyte of the EAD segment (cell number 140) for the patterns of Fig 12(A), 12(B) and 12(C) (blue, red, and black APs, respectively). We see an EAD in the black curve, but no EADs in the other two, which can be explained as follows. The gap-junctional current I_gap_ between the myocyte and fibroblast, in the early phase of the action potential, provides a transient outward current (resembling the transient outward potassium current I_to_) [43], where I_gap_ flows from the myocyte to the fibroblast. This reduces the plateau voltage of the AP; such a lowering of the plateau voltage is known to promote EADs, because the lowering of the plateau potential delays the activation of I_ks_ and enhances the inward calcium current I_CaL_[44]. This is why we find EADs in the black AP in Fig 13 (fibroblast pattern as in Fig 12(C)) but not in the blue AP (without fibroblasts as in Fig 12(A)). The red AP in Fig 13 (fibroblast pattern as in Fig 12(B)) has a low plateau voltage, like its black counterpart, but it does not show EADs, because it has a steeper repolarizing phase than the black AP. This difference in steepness follows from the qualitative difference in the plots of V_m_, along the cable in Fig 12(C) and 12(B). In Fig 12(C), the maxima in V_m_ lead to an influx of current from the regions with EAD myocytes into the region with fibroblasts; such an influx is absent in Fig 12(B), because the plot of V_m_ in the EAD segment has a flat plateau. Similarly, the peak-and-valley structure in the plot of V_m_ in the EAD segment in Fig 12(D) leads to EADs, and, thereby, to PVCs. Thus, the triggering of PVCs depends not only on the electrophysiological properties of fibroblasts and myocytes, but also on the spatial pattern of fibroblasts and EAD myocytes. Note that, although E_f_ = -35 mV is low enough to suppress EADs in an isolated EAD myocyte-fibroblast composite (Fig 9(B)), this does not necessarily imply the suppression of EADs (or PVCs) in tissue; this is an example where the single-cell level results cannot be used to predict the results at the level of tissue.

**Fig 14:**
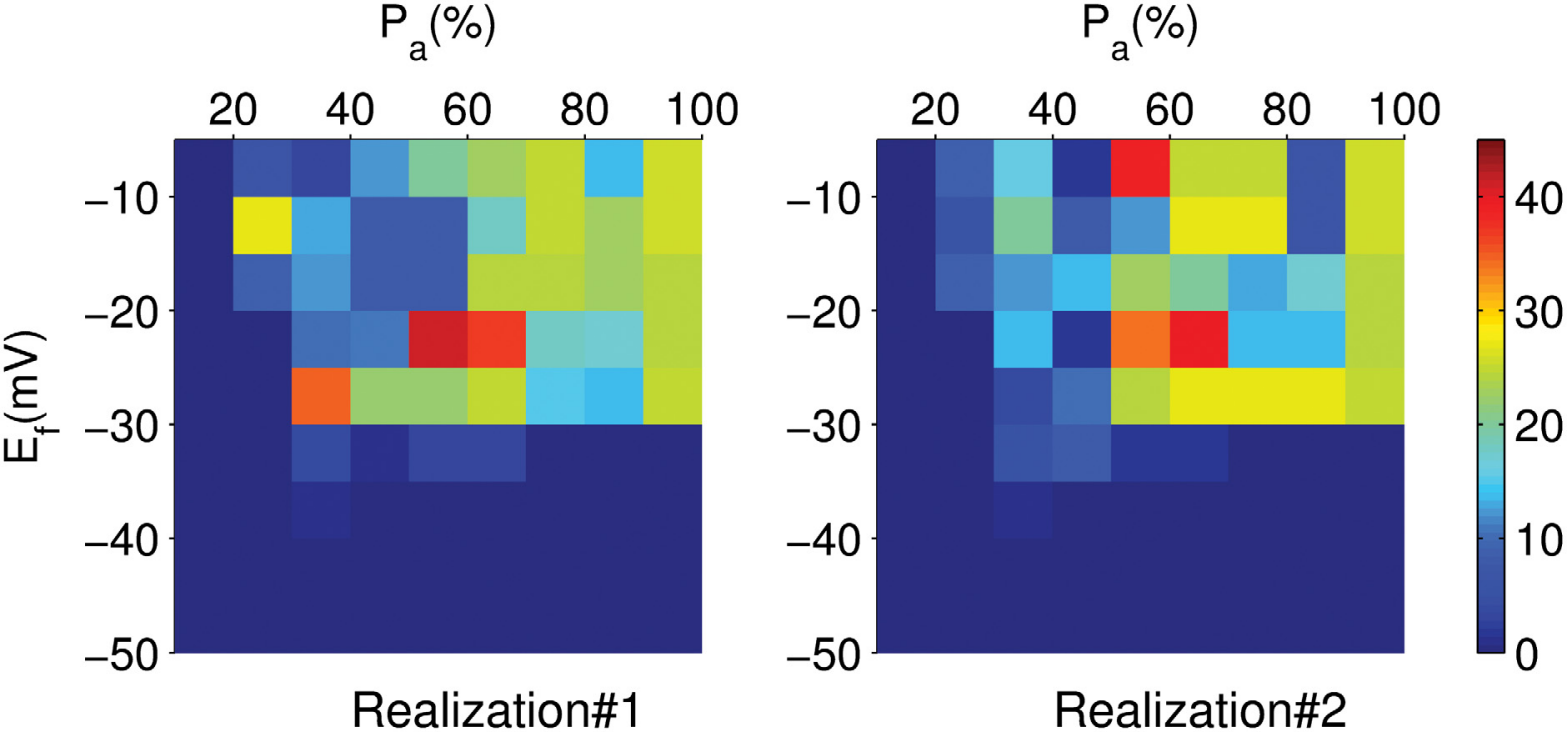
Number of PVC triggerings in the E_f_ − P_a_ plane in a 1D cable. Pseudocolor plots showing the different numbers N of PVC triggerings, indicated by different colors (see the colorbar), in the E_f_ − P_a_ plane for two different realizations of fibroblast distribution in a 1D cable with its EAD segment attached to fibroblasts randomly. P_a_ is the percentage of fibroblasts attached to the EAD segment. These plots illustrate the sensitive dependence of the number of PVCs on the distribution of fibroblasts.

We show in Fig 14, for a 1D cable with randomly attached fibroblasts in its EAD segment, the different numbers of PVC triggerings, indicated by different colors, in the E_f_-P_a_ plane, for two different realizations of the random configuration of fibroblasts. Fig 14 illustrates the sensitive dependence of PVC triggerings on E_f_, P_a_, and distribution pattern of fibroblasts on the EAD clump. There are no PVCs in the region with low values of E_f_ and P_a_.

Experimental evidence shows that the membrane conductance G_f_ of fibroblasts can depend on the voltage V_f_[45]. Therefore, we have carried out illustrating studies in which G_f_ has a nonlinear dependence on V_f_. In particular, we use two different values of G_f_, like in Refs. [46, 47], instead of using a constant value of G_f_. The value of G_f_ is set to 2 nS if V_f_ is below -20 mV, and 4 nS for V_f_ above -20 mV. Specifically, we find that high E_f_ values trigger PVCs (S1 Fig); reducing the myocyte-fibroblast coupling strength G_gap_ allows the EAD clump to regain its ability to trigger PVCs (otherwise it does not trigger PVCs at high values of G_gap_) (S2 Fig); and distributing the fibroblasts randomly assists the triggering of PVCs (S3 Fig).

## Spiral-Wave Dynamics

**Fig 15:**
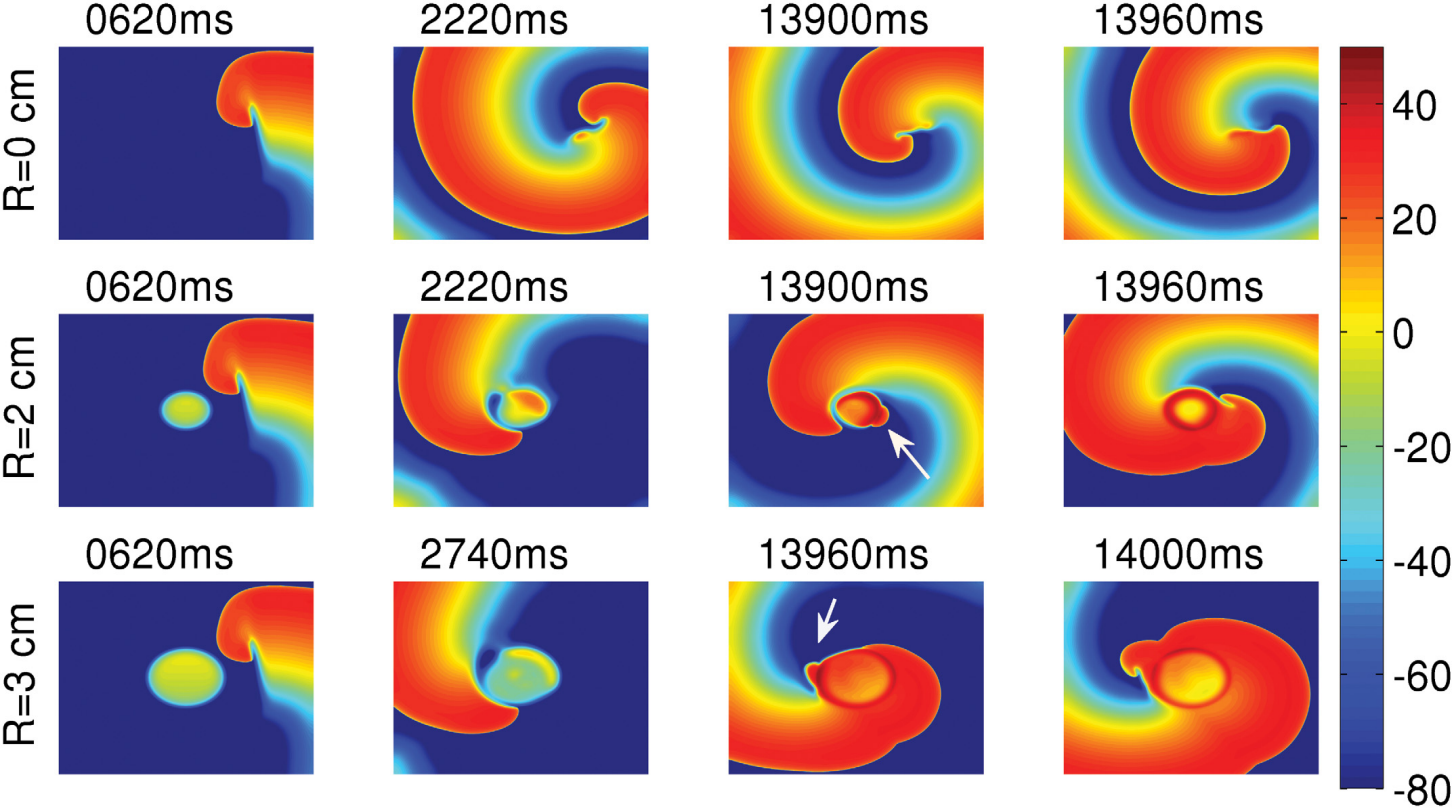
Pseudocolor plots of V_m_ illustrating spiral-wave dynamics in the presence of an EAD clump. The top panel shows the spatiotemporal evolution of a spiral in the absence of an EAD clump; the middle and bottom panels show the evolution of a spiral, initiated near clumps of radii R = 2 cm and R = 3 cm, respectively. In the presence of the clump, the spiral gets anchored to the clump, and the clump triggers excitations, indicated by white arrows at times 13900ms (middle panel) and 13960ms (bottom panel). These excitations block the progression of the spiral tip and, in turn, generate a new spiral tip.

**Fig 16:**
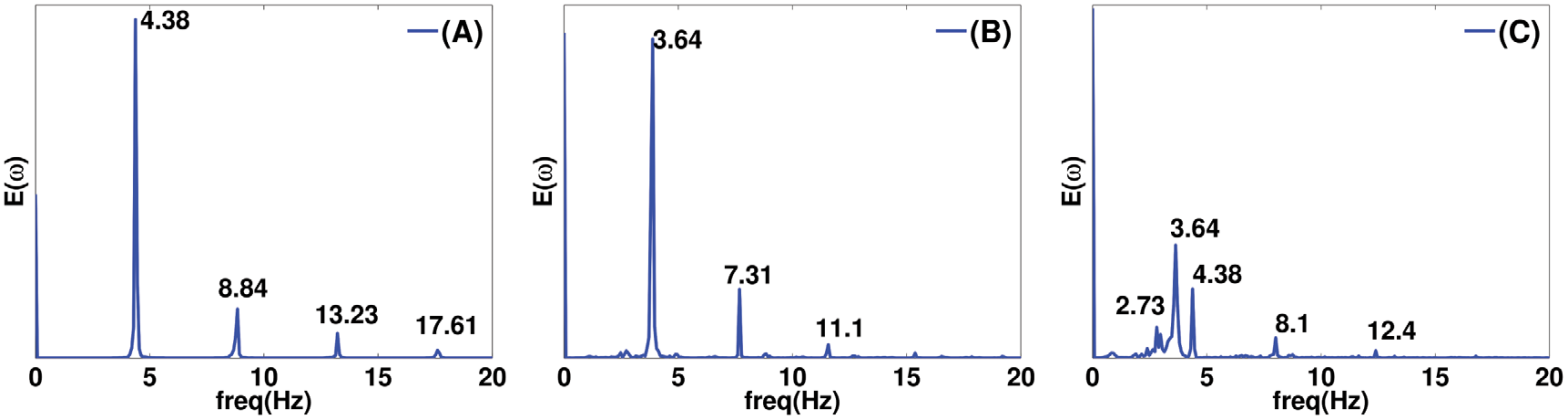
Averaged power spectra of the time series of V_m_ of Fig 15 from four representative points located near the four corners in the domain. (A) The power spectrum (E(*ω*)) of the free rotating spiral indicates a periodic motion with a fundamental frequency of 4.38 Hz. (B) The spectrum of the spiral anchored to the clump with R = 2 cm also indicates a periodic motion with the fundamental frequency reduced to 3.64 Hz. (C) The spiral anchored to the clump of radius R = 3 cm shows a quasiperiodic motion. The peaks in (C) can be indexed as n_1_ *ω*_1_ + n_2_ *ω*_2_, where n_1_ and n_2_ are integers, and *ω*_1_ ≃ 2.73 Hz and *ω*_2_ ≃ 3.64 Hz.

Here we study the spatiotemporal evolution of a spiral wave, which we initiate in the presence of an EAD clump. The size of the simulation domain is 19.2 × 19.2 cm^2^. We initiate the spiral near to the clump using S1–S2 cross-field protocol [48, 49]. The spiral is attracted by the clump and it gets anchored to the clump, as we show in the middle and bottom panels of Fig 15. Such an attraction of spiral waves has been observed in the presence of small ionic inhomogeneities in Ref. [50]. Our study illustrates the attraction of spiral waves also in the case of an EAD clump. In the presence of EAD clumps, the EADs trigger excitations, which can disrupt the motion of spiral tips, as we indicate by the white arrows in the middle and bottom panels of Fig 15 at times 13900 ms and 13960 ms, respectively. The excitations block the progression of the spiral tips and, in turn, generate new spiral tips (see S7 Video). Such excitations can destroy the periodicity of the spiral. If the radius of the clump is small, the periodicity of the spiral is maintained and the frequency of the spiral is reduced; but, if the radius is large, the excitations destroy the periodicity of the spiral. Fig 16(A), 16(B) and 16(C) show the averaged power spectra from four representative points, located near the four corners of the simulation domain, for the freely rotating spiral (Fig 15 top panel), the spiral attached to the clump of radius R = 2 cm (Fig 15 middle panel), and the one of radius R = 3 cm (Fig 15 bottom panel), respectively. This figure shows that the spiral attached to the clump of radius R = 2 cm still executes a periodic motion, with the frequency reduced to 3.64 Hz as compared to the freely rotating spiral with a frequency of 4.38 Hz. However, the temporal evolution of the spiral, attached to the clump of radius R = 3 cm, is quasiperiodic with two incommensurate frequencies; the peaks in Fig 16(C) can be indexed as n_1_ *ω*_1_ + n_2_ *ω*_2_, where n_1_ and n_2_ are integers and *ω*_1_ ≃ 2.73 Hz and *ω*_2_ ≃ 3.64 Hz.

## Discussion

Regional heterogeneities occur in the heart because of pathologies like myocardial ischemia [51, 52]; such heterogeneities may also exist inherently in ventricles in a normal heart [53, 54]. Thus, it is important to investigate how pathological cells in a localized region are arrhythmogenic. Here, we investigate the factors that can assist a cluster of EAD cells to trigger PVCs. First, we show that not all APs with EADs lead to PVCs. If the repolarization reserve of the myocyte is such that the EADs have a low amplitude and decaying oscillations as in type-II APs (Fig 2(B)), the EAD clumps cannot trigger PVCs. Second, a clump of EAD myocytes triggers PVCs only after a particular threshold size, characterized here by radius R; and the number N of PVC triggerings increases, roughly speaking, with R; however, the plot of N versus R depends sensitively on parameters such as the pacing cycle length PCL. Third, we show that a reduction in the coupling strength D, between the EAD cells, also assists in triggering PVCs, because, if D is low, more cells in the clump retain their ability to trigger EADs, and, thereby, facilitate the triggering of PVCs. Fourth, we show that fibrosis aids in the triggering of PVCs. The presence of fibrosis, i.e., the interspersion of collagen deposits from fibroblasts (modelled as inexcitable obstacles), reduces the effective number of neighboring myocytes, and, thereby, lowers the local source-sink mismatch, which thus aids in the triggering of PVCs. We show that N increases with the percentage of fibrosis P_f_, up to 40%, and then decreases as P_f_ increases, indicating a trade-off between the percentage of fibrosis and the number of EAD myocytes for PVC triggerings. This finding, that the increase of the percentage of fibrosis up to an intermediate value facilitates the triggering of PVCs, agrees with the experimental study conducted on intact rat hearts by Morita, *et al.* [15].

*In vitro* studies show that, the formation of gap-junctional coupling between fibroblasts and myocytes can be arrhythmogenic, as the coupling can promote EADs [55] or induce ectopic activity [56]. We show how the gap-junctional coupling between the fibroblasts and the EAD myocytes modulates the PVC-triggering ability of the EAD clumps. We demonstrate that the resting membrane potential E_f_ of the fibroblasts plays an important role in modulating the PVC-triggering ability of the EAD clump. The fibroblasts either assist or impede the triggering of PVCs, depending on the value of E_f_. In an EAD myocyte-fibroblast composite, low values of E_f_ (< -40 mV) suppress EADs and hence suppress PVCs; high values of E_f_ (E_f_ > -32 mV) enhance EADs and hence promote PVCs. Another important factor is the gap-junctional coupling strength (G_gap_) between the fibroblasts and myocytes, the conduit for the fibroblast’s influence on the electrophysiology of EAD myocytes.

We also find that the triggering of PVCs by an EAD clump, with fibroblasts attached, depends sensitively on the distribution pattern of the fibroblasts. In an EAD clump, which is attached to fibroblasts distributed inhomogeneously, the presence of local minima and maxima in V_m_, associated with the inhomogeneous distribution of fibroblasts, promotes PVCs. In such EAD clumps, we observe the triggering of PVCs, even if E_f_ is low enough to suppress EADs in the isolated EAD myocyte-fibroblast composite. This result is an example of a case where a single-cell result cannot be extrapolated naïvely to predict tissue-level results. To conclude the part of our study of EAD clumps with fibroblasts, we have shown that the proliferation of fibroblasts in tissue can influence the PVC-triggering ability of an EAD clump in two ways: 1) By depositing collagens in the tissue, which in turn reduces local source-sink mismatch and promotes PVCs; 2) by forming gap-junctional coupling with the myocytes, which either promotes or impedes PVCs, depending on the electrical properties of the fibroblasts and the distribution pattern of the fibroblasts.

Finally, we have shown, with a few representative simulations, how an EAD clump can interact with a spiral, which is initiated near the clump. We observe that the spiral gets anchored to the clump, and the excitations triggered by the EAD clump annihilate the spiral tip and also, in turn, generate a new spiral tip. We find that, if R is small, the spiral rotates around the clump in a periodic manner, but, if R is large, the spiral rotates quasiperiodically.

A disease like heart failure is associated with the features that we have investigated in this paper, namely, the promotion of EADs [24–27], a reduced coupling strength because of the reduction in the expression of the gap-junctional protein connexin43 [22, 23], and also fibrosis [20, 21]. Such failing hearts can thus be susceptible to premature ventricular complexes (PVCs). Therefore, our detailed *in silico* study of the various factors that promote PVCs in a mathematical model for cardiac tissue help us in understanding and characterizing the risk of fatal cardiac diseases associated with PVCs.

We end our discussion with some limitations of our study. Our tissue simulations are restricted to 2D domain without tissue anisotropy. Such tissue anisotropy can affect the triggering of PVCs. An earlier study by Sato, *et al.* [10] has investigated the effects of tissue anisotropy on the propagation of EADs in 2D tissue; however, simulations in 3D tissue with fiber architecture, or in anatomically-realistic heart can improve our understanding of the propagation of EADs, manifesting as PVCs, in mammalian hearts. We have used an isotropic monodomain representation of cardiac tissue; our study needs to be extended to other tissue models, such as those that use bidomain representations [57], or novel fractional descriptions accounting for tissue microstructure [58]. The maximum fibroblast-myocyte coupling ratio we use in our study is equal to 1, which means that the maximum number of fibroblasts that is connected to a myocyte is 1; we have not investigated the effects of higher fibroblast-myocyte ratio on the triggering of PVCs. However, an earlier study by Morita et al. [15] has found that PVC triggering is prominent at intermediate values of fibroblast-myocyte ratio. Also, the fibroblasts, in our study, are modelled as passive cells by using the model given by MacCannell, *et al.* [34]. However, there is evidence that fibroblasts can also behave as active cells [34, 59, 60]. A detailed study of the effects of such active fibroblasts on EAD myocytes and their influence on the triggering of PVCs lies beyond the scope of the present investigation.

## Supporting Information

S1 Video Dependence of PVC triggering on the repolarization reserve. Video showing the triggering of PVCs, by an EAD clump, which yields type-I AP (Fig 2(A)). The EAD clump with type-II AP (Fig 2(B)) does not trigger PVCs. The clumps have the same radii R = 2.4 cm. For this video, we use 10 frames per second with each frame separated from the succeeding frame by 20ms in real time.
(MPEG)

S2 Video Dependence of PVC triggering on the size of an EAD clump. Video showing the triggering of PVCs by an EAD clump with radius R = 2.4 cm, and the absence of PVCs in the case of the smaller clump with radius R = 2.2 cm. For this video, we use 10 frames per second with each frame separated from the succeeding frame by 20ms in real time.
(MPEG)

S3 Video Dependence of PVC triggering on the coupling strength D between the EAD cells. Video showing PVC triggering by an EAD clump, radius R = 2 cm, when the coupling strength between the EAD cells in the clump is reduced (reducing the diffusion constant D). At very low coupling strength (D = 0.2xD_o_), the clump supports spirals of small wavelengths, which increases the PVC-triggering rate. For this video, we use 10 frames per second with each frame separated from the succeeding frame by 20ms in real time.
(MPEG)

S4 Video Dependence of PVC triggering on the percentage of fibrosis. Video showing PVC triggering by an EAD clump, radius R = 2 cm, when the percentage of fibrosis P_f_ = 15%. At low percentage (P_f_ = 10%) the clump does not trigger PVCs. For this video, we use 10 frames per second with each frame separated from the succeeding frame by 20ms in real time.
(MPEG)

S5 Video Dependence of PVC triggering on the value of the resting membrane potential E_f_ of the fibroblasts. Video showing PVC triggering by an EAD clump, radius R = 2.2 cm, when we attach the clump to fibroblasts with E_f_ = -30 mV. If the value of E_f_ is reduced to -35 mv, the EAD clump does not trigger PVCs. For this video, we use 10 frames per second with each frame separated from the succeeding frame by 20ms in real time.
(MPEG)

S6 Video Dependence of PVC triggering on the coupling strength G_gap_ between the myocytes and fibroblasts. Video showing the suppression of PVC triggering of an EAD clump with radius R = 2.4 cm when attached to fibroblasts of E_f_ = -35 mV with G_gap_ = 8 nS. When G_gap_ is reduced to 1 nS, the EAD clump regains its ability to trigger PVCs. For this video, we use 10 frames per second with each frame separated from the succeeding frame by 20ms in real time.
(MPEG)

S7 Video Spiral-wave dynamics in the presence of an EAD clump. Video showing how the presence of an EAD clump anchors the spiral to the clump and can disturb the periodicity of the motion of the spiral. The top panel shows the spiral motion in the absence of an EAD clump. The bottom-left and the bottom-right panels show the spiral motion in the presence of EAD clumps of radii R = 2 cm, and R = 3 cm, respectively. The spiral anchored to the small clump (R = 2 cm) rotates in a periodic motion, whereas the spiral anchored to the large clump (R = 3 cm) does not exhibit a periodic motion, but rather rotates in a quasiperiodic manner (cf. Fig 16). The EAD clumps trigger excitations that block the progression of the spiral tip and, in turn, generate a new spiral tip. For this video, we use 10 frames per second with each frame separated from the succeeding frame by 20ms in real time.
(MPEG)

S1 Fig The dependence of PVC triggering on E_f_ of the fibroblasts (with a nonlinear G_f_ (see text, page number 9)) in an EAD myocyte-fibroblast clump. Pseudocolor plots of V_m_ showing that an EAD myocyte-fibroblast clump, with R = 2.2 cm, triggers PVCs if E_f_ = -30 mV (bottom panel), but is unable to trigger PVCs if E_f_ is reduced to -40 mV (top panel).
(EPS)

S2 Fig The dependence of PVC triggering on G_gap_ (where the fibroblasts have a nonlinear G_f_). Pseudocolor plots of V_m_ showing that the EAD clump, with radius R = 2.4 cm, loses its ability to trigger PVCs when coupled to fibroblasts of E_f_ = -40 mV and G_gap_ = 8 nS (top panel), but retains its ability to trigger PVC, if G_gap_ is reduced to 0.3 nS (bottom panel).
(EPS)

S3 Fig The triggering of PVCs by an EAD clump with randomly attached fibroblasts (with a nonlinear G_f_). Pseudocolor plots of V_m_ showing the triggering of PVCs by the clump with 40% random distribution of fibroblasts.
(EPS)
