# Correction: A Computational Study of the Factors Influencing the PVC-Triggering Ability of a Cluster of Early Afterdepolarization-Capable Myocytes

**DOI:** 10.1371/journal.pone.0148663

**Published:** 2016-02-05

**Authors:** The PLOS ONE staff

Figs 5 and 6 are incorrect. The figure captions of Figs 5 and 6 are swapped. The publisher apologizes for the error. The authors have provided the corrected versions here.

**Fig 5:**
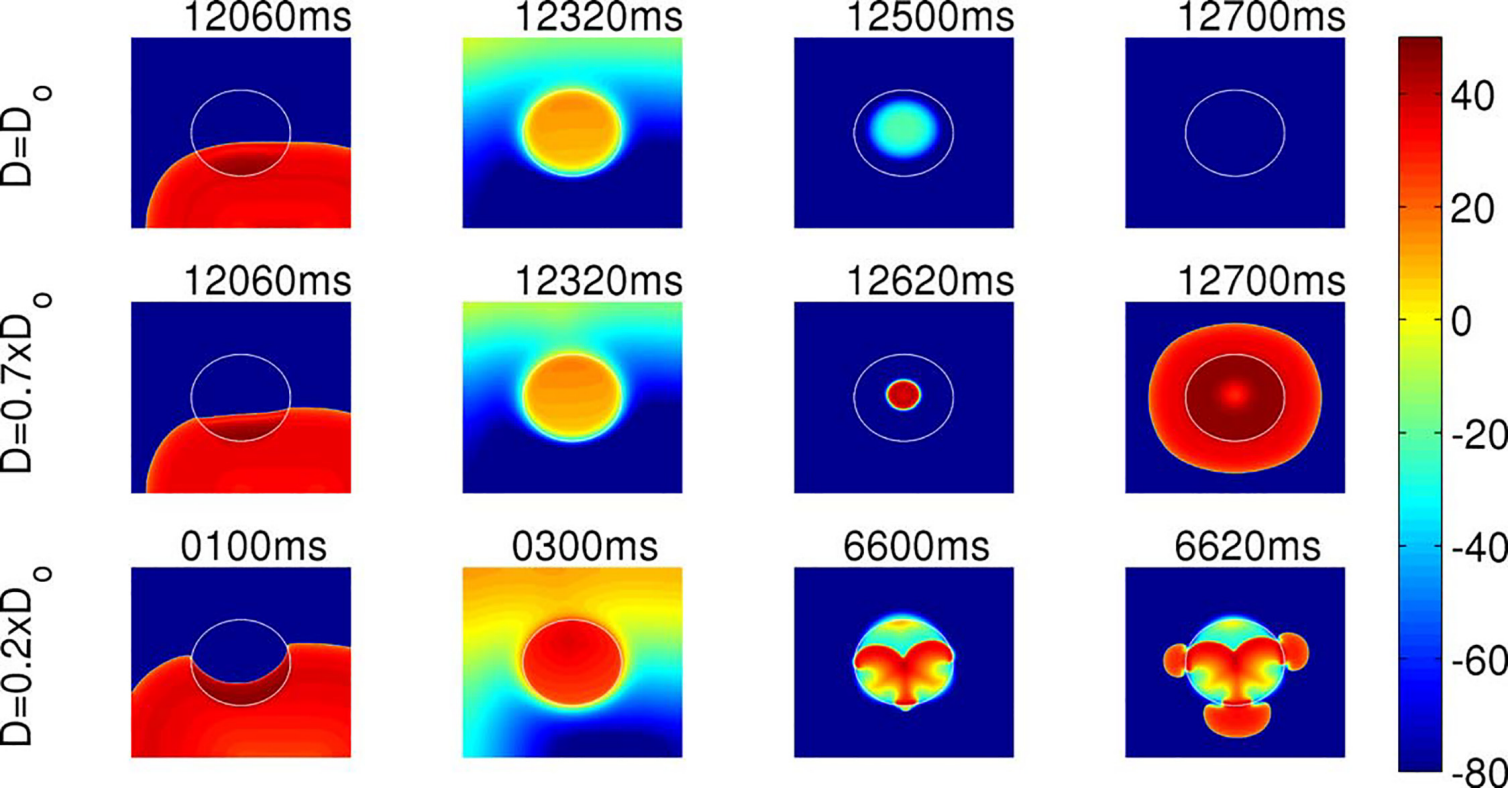
The dependence of the triggering of PVCs on the coupling strength of the EAD clump. The clumps in the top, middle, and the bottom panels have radii R = 2 cm. The clump with D = Do (top panel) does not trigger PVCs, whereas the clumps with D = 0.7xDo, (middle panel) and D = 0.2xDo (bottom panel) trigger PVCs. The clump with D = 0.2xDo supports small-wavelength spirals inside the clump as shown at times 6600 ms and 6620 ms.

**Fig 6:**
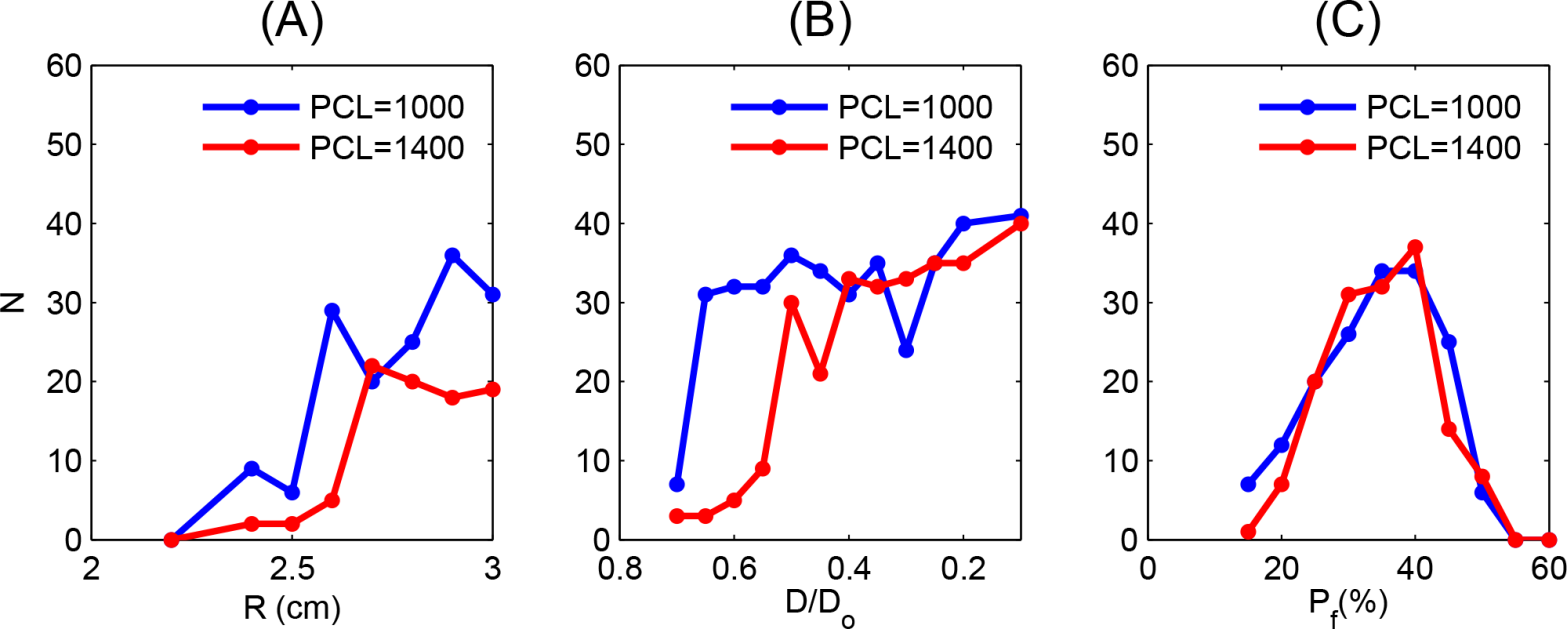
Dependence of the number N of PVC triggerings on the radius R, normalized coupling strength D/Do, and the percentage of fibrosis Pf. The blue and red curves are for PCL = 1000 ms and 1400 ms, respectively. (A) Plots of N versus R, which show that the number of PVCs increases roughly with R. (B) Plots of N versus D/Do. The number of PVC triggerings increases initially with the reduction of D/Do, but saturates roughly when D/Do ≲ 0.4 and ≲0.65 for PCL = 1000 and 1400 ms, respectively. (C) Plots of N versus the percentage of fibrosis Pf show a maximum at Pf ≃ 40%. A comparison of the plots in (A), (B), and (C) for the two different values of PCL, namely, PCL = 1000 ms and PCL = 1400 ms, shows that these plots depend sensitively on PCL.
